# LightGBM: accelerated genomically designed crop breeding through ensemble learning

**DOI:** 10.1186/s13059-021-02492-y

**Published:** 2021-09-20

**Authors:** Jun Yan, Yuetong Xu, Qian Cheng, Shuqin Jiang, Qian Wang, Yingjie Xiao, Chuang Ma, Jianbing Yan, Xiangfeng Wang

**Affiliations:** 1 National Maize Improvement Center, Department of Crop Genomics and Bioinformatics, College of Agronomy and Biotechnology, China Agricultural University, Beijing, 100193 China; 2 Key Laboratory of Biology and Genetics Improvement of Maize in Arid Area of Northwest Region, Ministry of Agriculture, Northwest A&F University, Shaanxi, China; 3 National Key Laboratory of Crop Genetic Improvement, Huazhong Agricultural University, Wuhan, 430070 China

**Keywords:** Genomic prediction, Genomic selection, Machine learning, Ensemble learning, Maize, Crop breeding, LightGBM, rrBLUP

## Abstract

LightGBM is an ensemble model of decision trees for classification and regression prediction. We demonstrate its utility in genomic selection-assisted breeding with a large dataset of inbred and hybrid maize lines. LightGBM exhibits superior performance in terms of prediction precision, model stability, and computing efficiency through a series of benchmark tests. We also assess the factors that are essential to ensure the best performance of genomic selection prediction by taking complex scenarios in crop hybrid breeding into account. LightGBM has been implemented as a toolbox, CropGBM, encompassing multiple novel functions and analytical modules to facilitate genomically designed breeding in crops.

**Supplementary Information:**

The online version contains supplementary material available at 10.1186/s13059-021-02492-y.

## Background

The rapid advancement of genotyping technology has promoted the integration of genomic prediction into modern breeding programs for both animals and crops [1–5]. Predictive models built using various computational methodologies have been employed to facilitate data-driven decision-making in terms of selecting breeding materials and designing hybridization schemes [6, 7]. As most traits subject to selective breeding are determined by quantitative trait loci (QTLs), genomic selection (GS) has been validated as an effective approach utilizing whole-genome variations to build genomic prediction models without prior characterization of trait-associated genes [8, 9]. Among existing GS tools, ridge regression BLUP (rrBLUP) is a state-of-the-art method that uses a linear mixed-effect model to deduce the genomic kinship of breeding materials and marker effects for phenotype prediction [10].

GS for crops is associated with many more challenges than that for animals [11]. The main factor in a GS project for crops is the phenotyping expense incurred in constructing a training set. In maize breeding, which mainly utilizes heterotic effects, GS predicts the hybrid performance of filial one (F_1_) generated from the crossings of inbred lines from different heterotic pools. An incomplete diallel cross of two parental pools each containing 1000 lines can theoretically generate one million F_1_ combinations, from which only ~ 15% are planted to measure the phenotypes for training model to predict the rest ~ 85% [12]. With the application of doubled haploid (DH) and speed breeding (SB) technologies in the seed industry, the productivity of inbred lines per year has been substantially increased [3–5]; however, screening on tens of thousands of lines largely relies on genotypes [2]. Hence, novel GS methods capable of coping with large sample sets are in high demand. Moreover, the genetic compositions of crops are complex because of the diverse genetic origins and frequent crossings among germplasms. This may cause population stratification, which significantly influences model stability [12]. Finally, heterosis has been hypothesized as a result of non-additive effects involving not only epistatic interactions among QTLs, but also environmental effects [13, 14]. Therefore, linear models lack the capability to fully infer all the forms of genetic effects contributing to heterosis; nonparametric methods suitable for predicting the hybrid performance of F_1_s are desired for crop breeding [15–17].

Machine learning (ML) has been widely employed in Big Data analytics. It can automatically learn data patterns and optimize model parameters [18, 19]. The gradient boosting (GB) is a member of the ensemble learning paradigm, and its underlying principle involves assembling multiple weak learners to establish a strong model; thus, its predictability is significantly better than that of a single model [20]. As GB is a tree-based ensemble algorithm, it is particularly capable of deciphering categorical features. Employment of GB algorithm for GS has been previously tested in both cattle and plants, in which GB showed better performance than random forest (RF), artificial neural network (ANN), and convolutional neural network (CNN) [6, 19, 21]. Although the GB and RF both adopt the ensemble paradigm, GB outperformed RF, likely because the RF builds independent trees and averages the results from all the trees as the final prediction, whereas the GB builds trees by boosting iterations [22, 23]. In each iteration, the current tree is built based on the last tree in which the difference between the predicted and actual values is computed and set as the predictive goal for the current tree. Subsequently, the difference is gradually minimized after hundreds of iterations, and the results from all the trees are summed as the final prediction. In comparison with ANN and CNN, GB adopts a different strategy of feature extraction. GB first traverses all the features to select important nodes when building trees, and the prediction is only based on features with a high effectiveness in classifying the samples; or, in other words, it simultaneously accomplishes feature selection and prediction. Most importantly, the selected features remain in their original form without any weight applied to them. When applying ANN or CNN for genomic prediction, the algorithm performs feature extraction by first estimating a weight for each SNP and then computing a reformed, grand feature based on the sum of the weighted features to represent a set of neighboring SNPs within a predefined genomic region [24, 25]. If the region contains too many SNPs unrelated to the predicted trait, the effectiveness of the grand feature might be significantly attenuated. Thus, overly excessive features may significantly reduce the precision and stability of a neural network and may lead to model training failure because of exploding gradients.

One of the GB variants — LightGBM (light gradient boosting machine) — developed by Microsoft has demonstrated superior performance in coping with extremely large, structured datasets with an ultra-high training speed [23]. In this work, we evaluated the capability of LightGBM for GS prediction in maize breeding. Our results demonstrate the extraordinary performance of LightGBM in terms of its precision, model stability, and computing efficiency. The LightGBM algorithm was implemented as a toolbox named CropGBM (Genomic Breeding Machines for Crops) to facilitate genomically designed crop breeding.

## Results

### Training and testing samples

**Fig. 1:**
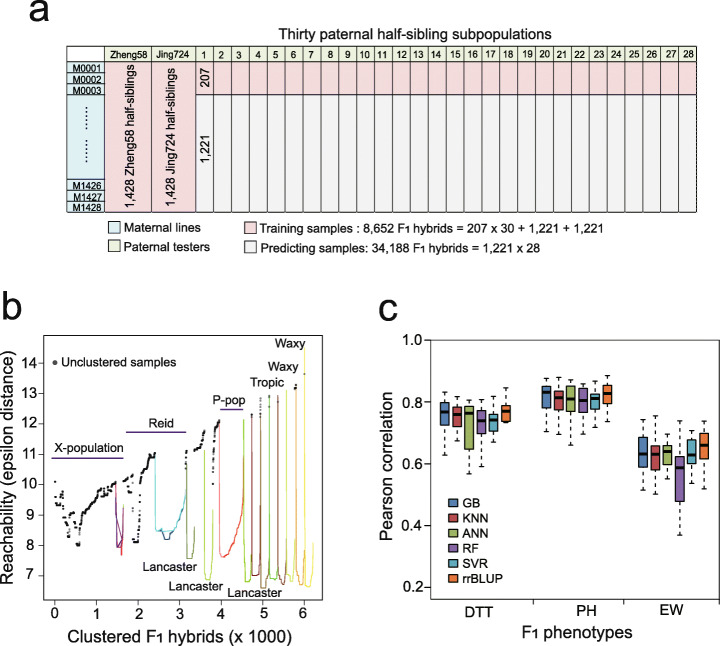
Evaluation on the five basal machine learning (ML) models. **a** Data structure of the 42,840 F_1_s generated from the crossings of 1428 maternal lines and 30 paternal testers. The training samples include a total of 8652 F_1_s (207 maternal lines × 28 paternal testers, 1428 maternal lines × Zheng58, and 1428 maternal lines × Jing724). **b** Classification of the 6210 F_1_s by the OPTICS clustering algorithms. **c** Comparison of the five commonly used ML methods, namely support vector regression (SVR), random forests (RF), artificial neural network (ANN), k-nearest neighbor (KNN), and gradient boosting (GB) algorithms. GB exhibits precision and stability equivalent to that of rrBLUP

The dataset for model evaluation included 8652 samples of F_1_ hybrid maize with measured phenotypes of days to tasseling (DTT), plant height (PH), and ear weight (EW), generated from the crossings of a maternal pool and a panel of 30 paternal testers following a North Carolina-II design (Methods) [26]. The maternal pool was a previously reported CUBIC (Complete-diallel design plus Unbalanced Breeding-like Inter-Cross) population containing 1428 inbred lines, developed from 24 elite founder lines representing local-adaptive alleles [27]. The paternal pool contained 30 tester lines covering six major heterotic groups, mostly improved oversea germplasms representing foreign advantageous alleles. The population is thus composed of thirty sets of paternal half-sibling subpopulations (called F_1_s for short) exhibiting diverse patterns of heterosis effects of hybrid maize. The data structure of the thirty F_1_ subpopulations is illustrated for designing different predictive frameworks to objectively evaluate the model precision and stability (Fig. 1a). The 8652 samples contained two complete F_1_ populations consisting of 2856 hybrids between the 1428 maternal lines and the 2 paternal tester lines Zheng58 and Jing724. Crossings of the 207 maternal lines and 30 paternal testers generated 6210 F_1_s exhibiting strong population stratification in genotypes, due to the diverse genetic origins of the maternal founders and paternal testers (Fig. 1b, Additional file 1: Fig. S1). To remove systems bias in phenotypic variations between subpopulations, the phenotypic values were normalized to *z*-scores within each F_1_ population, for which relative rankings were used to represent the absolute values (Additional file 1: Fig. S2). As a set of non-redundant features is crucial for ML to avoid dimension explosion, 32,559 haplotypic tag single-nucleotide polymorphisms (SNPs) evenly distributed in the genome were used as genotype features (Methods).

### Evaluation on basal ML models

To ensure an objective assessment of ML methods in GS, we followed the procedure of constructing an ML system covering feature selection, model selection, model evaluation, model optimization, feature importance analysis, model interpretation, and finally, software benchmark testing. We first performed a series of benchmark tests between rrBLUP and other 15 previously published GS tools using the dataset of the 1428 maternal lines (Methods). rrBLUP exhibited superior prediction precision and computing efficiency, and it was then selected as the representative of statistical models to compare with ML methods (Additional file 1: Fig. S3). As different ML algorithms may be suitable for different predictive goals and data features, we selected five commonly used basal ML models for the initial evaluation and compared them with rrBLUP, namely support vector regression (SVR), random forests (RF), artificial neural network (ANN), k-nearest neighboring (KNN), and gradient boosting (GB) regression tree. Evaluation of the five MLs models were under the optimal hyperparameter tuned by the function of grid search (Additional file 1: Table S1). The 6210 F_1_s with field-measured phenotypes were mainly used for evaluating the model precision. Cross-validation (CV) was performed using the following framework repeated 30 times to generate a distribution of the precision (*r*, Pearson’s correlation coefficient) for each method: namely 29 subpopulations of F_1_s as training samples to predict the rest one using the 32,559 SNP as features. As the precisions of GB and rrBLUP were equivalent and both outperformed other four ML methods (Fig. 1c), GB was then selected for a further comparison with rrBLUP.

**Fig. 2:**
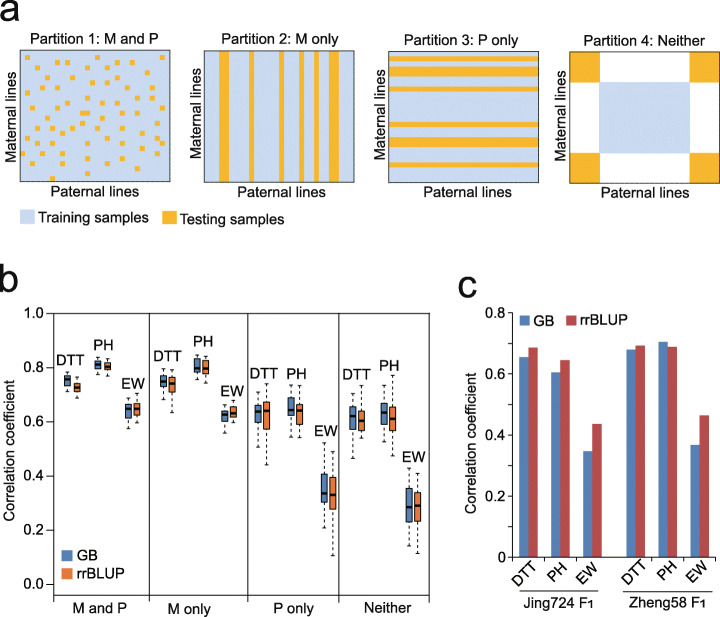
Evaluations of GB and rrBLUP precision on different predictive frameworks. **a** Schematic illustration of the four ways partitioning the training and testing samples. M and P: training samples cover the maternal and paternal genotypes of testing samples. M only: training samples only cover the maternal genotypes of testing samples. P only: training samples only cover the paternal genotypes of testing samples. Neither: training samples cover neither of the maternal and paternal genotypes of the testing samples. **b** Evaluations of GB and rrBLUP precision on the four different predictive frameworks. **c** Evaluations of GB and rrBLUP precision between two fully genotyped F_1_ populations. Left three columns: 1428 Zheng58 F_1_s as training samples to predict Jing724 F_1_s; Right three columns: 1428 Jing724 F_1_s as training samples to predict Zheng58 F_1_s

The predictive frameworks with different ways of partitioning the samples may influence model stability, and inappropriate partitions may lead to incorrect overfitting because of population stratification. Particularly for the hybrid breeding of maize, which mainly relies on the crossing between distantly related heterotic groups, the predictive framework is a critical factor in ensuring model stability. To address this issue, the training-to-testing sample ratio was set to 5:1 using four different frameworks: when the training samples covered the genotypes of both maternal and paternal (M and P) siblings, only maternal (M only) siblings, only paternal (P only) siblings, and neither parental (Neither) siblings in the testing samples (Fig. 2a). As shown in Fig. 2b, the training samples covering both parental genotypes exhibit the highest precision, followed by those covering only maternal genotypes; precisions greatly drop when the training samples only cover paternal genotypes or neither of parental genotypes. Particularly for EW, the precision drops nearly twofold when neither parents are covered in the training samples. We then evaluated the model stability on two sets of fully phenotyped Zheng58 and Jing724 F_1_s, using one population to train the models and predict the other one. The precision of rrBLUP was slightly better than that of GB, likely because the maternal lines are closely related siblings from the same CUBIC population with explicit pedigree (Fig. 2c).

### Evaluation on the LightGBM model

**Fig. 3:**
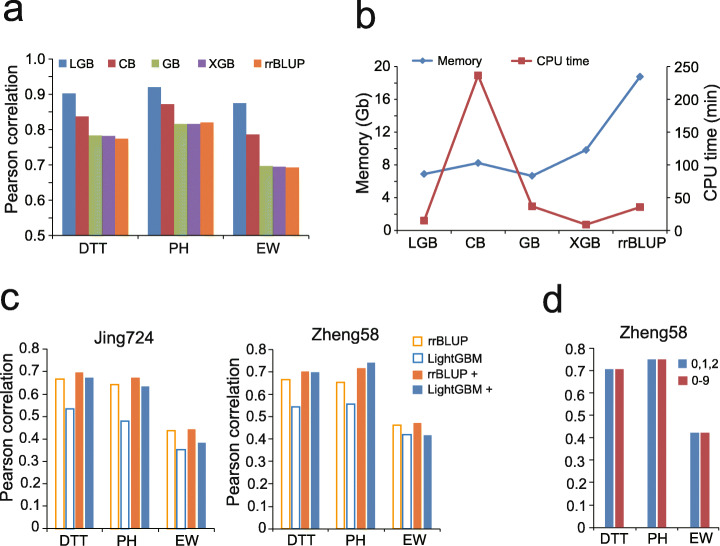
Comparison between LightGBM and rrBLUP. **a** Comparison of three variants of GB algorithms, namely LightGBM (LGB), XGBoost (XGB), and CatBoost (CB), with GB and rrBLUP in terms of the model fitting ability. **b** Comparison between the five methods in terms of the computing time and memory usage. **c** Improvement in the model precision of rrBLUP (rrBLUP+) and LightGBM (LightGBM+) via the addition of parental phenotypes as additional features. Left panel: 6210 F_1_s + 1221 Zheng58 F_1_s as training samples to predict 1221 Jing724 samples. Right panel: 6210 F_1_s + 1221 Jing724 F_1_s as training samples to predict 1221 Zheng58 F_1_s. **d** Use of two coding schemes (0, 1, 2 and 0–9) for converting genotypic characters to numeric features to compare the precision of LightGBM. Predictive framework: 6210 F_1_s + 1221 Jing724 F_1_s as training samples to predict 1221 Zheng58 F_1_s

An initial evaluation of the basal MLs indicated that ensemble learning with the GB paradigm is the optimal method, making it worthy of further evaluation and comparison with the optimized algorithms. Three more GB variants, namely LightGBM, XGBoost (eXtreme Gradient Boosting), and CatBoost (Categorical Boosting), were compared with GB and rrBLUP in parallel. We utilized the phenotyped 8652 F_1_s as the training samples to first evaluate the fitting ability of the five models. As shown in Fig. 3a and b, LightGBM has the highest fitting ability compared to the other four methods, and consumes only one third of the memory used by rrBLUP. Although CatBoost ranks as the second method in terms of precision, it consumes almost 100-folds of CPU time than LightGBM.

To evaluate the model stability of rrBLUP and LightGBM, we utilized a global collection of a core germplasm containing 527 lines of tropical, subtropical, and temperate maize [28] (Additional file 1: Fig. S4). The 527 lines were crossed with Zheng58 and Mo17 to generate two F_1_ populations with field-measured phenotypes of DTT, PH, and EW. Model evaluations were carried out using six predictive frameworks (E1 to E6) to compare LightGBM and rrBLUP (Additional file 1: Fig. S5a). While reciprocally using the 1428 Zheng58 (Jing724) F_1_s as the training samples to predict the 1428 Jing724 (Zheng58) F_1_s (E1 and E2), rrBLUP showed slightly higher precision than LightGBM. As for the reciprocal evaluations (E3 and E4) between Zheng58 and Mo17 F_1_s, LightGBM outperformed rrBLUP overall for DTT and PH (Additional file 1: Fig. S5b). While equally partitioning each F_1_ population to use one half of the samples as the training set to predict the other half (E5 and E6), the model precision could be improved; LightGBM was slightly better than or equal to rrBLUP. These results indicate that under different genetic backgrounds, the two methods produce relatively equivalent precision results when using different predictive frameworks.

Subsequently, we enlarged the dataset by combining the 6210 F_1_s reciprocally with the 1221 Zheng58 (Jing724) F_1_s as the training samples to predict the 1221 Jing724 (Zheng58) F_1_s. Because of the significant increase in the genetic complexity of the 7421 training samples, the influence of population stratification must be considered. One possible solution is to include parental phenotypes as additional features. As expected, the inclusion of additional features significantly improves the precision of LightGBM, from 0.538 to 0.686 (DTT), 0.518 to 0.687 (PH), and 0.386 to 0.400 (EW), on average for the two F_1_ populations (Fig. 3c).

When using any GS tool, the genotypic characters must be converted to numeric features. A common way of conversion is based on the allele frequency of SNP in a designated population, normally using 0, 1, and 2 to represent homozygous major alleles, heterozygous alleles, and homozygous minor alleles, respectively. Because the coding scheme for multi-allelic SNPs is complicated for rrBLUP, usually only bi-allelic SNPs are retained in the genotype file. If the species has a polyploid genome, the coding scheme is even more complicated than that of a diploid genome. LightGBM may employ an alternative coding scheme which converts the ten states of genotypic characters to ten types of numeric features for a diploid species (Methods). Once the conversion is determined for a fixed coding scheme, LightGBM may be directly applied for GS. On the same dataset shown in Fig. 2c, LightGBM generated exactly the same predicted values when using the two coding schemes (Fig. 3d). Therefore, the novel coding scheme of LightGBM may greatly make the step of processing genotype data easier, especially convenient for the application of GS on a polyploid species.

### Sampling rate of training data determines GS precision

**Fig. 4:**
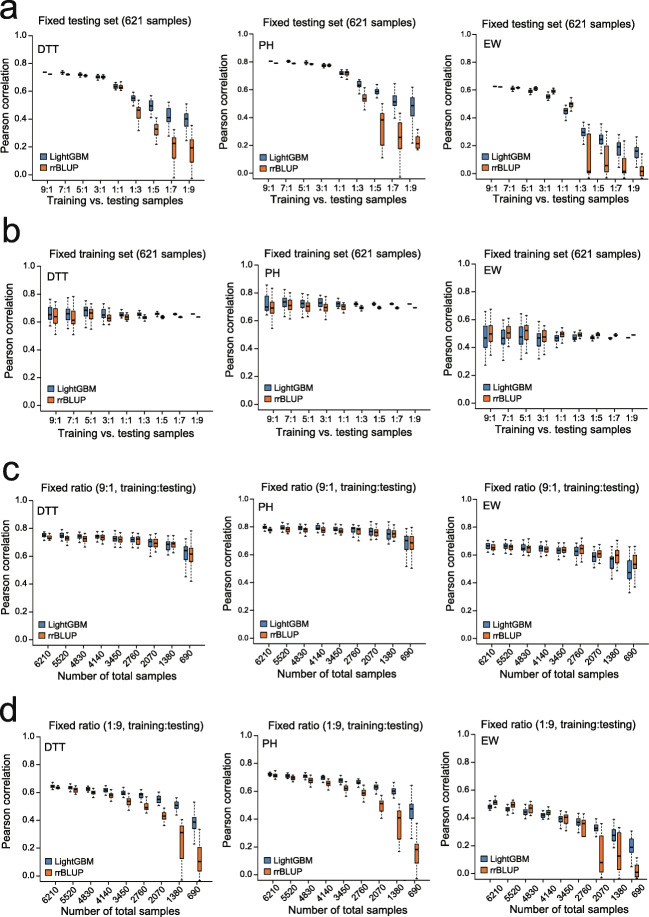
Sampling rate of training data determines baseline precision. **a** The number of 621 testing samples remained fixed, and nine sets of training samples with different numbers were randomly selected from the rest 5589 samples. Precisions of rrBLUP and LightGBM were then compared at the nine ratios of training and testing samples. **b** The number of 621 training samples remained fixed, and nine sets of testing samples with different numbers were randomly selected from the rest 5589 samples. **c** The ratio of 9:1 of training-to-testing samples remained fixed, and different numbers of samples were randomly selected from the total 6210 samples. Precisions of rrBLUP and LightGBM were then compared at the nine sets of training and testing samples. **d** The ratio of 1:9 of training-to-testing samples remain fixed, and different numbers of samples were randomly selected from the total 6210 samples

In a commercial pipeline of maize single-hybrid breeding, hybridization of inbred lines selected from maternal and paternal pools may generate large amount of F_1_ combinations. Breeders usually select 10 to 15% of the theoretical combinations to measure phenotypes as a training set, and predict the rest 85 to 90% via GS [12]. This may result in insufficient coverage of the genotypes of testing samples by a small proportion of training samples. Thus, estimation of GS precision under different sampling rates of training data is essential to ensure ideal efficiency of line selection for a population. To address this issue, we performed a series of benchmark tests on the population of 6210 F_1_s, to compare rrBLUP and LightGBM under four scenarios by setting different ratios of training and testing samples. In scenario 1, the size of testing set (621 sample) holds constant and the number of training samples gradually decreases from 5589 to 62, in order to set 9 ratios of training vs. testing samples. As shown in Fig. 4a, the ratio of 1:1 appears to be a turning point from which precision and stability of both methods begin to drop, but LightGBM significantly outperforms rrBLUP. This result indicates the advantage of LightGBM over rrBLUP when the sampling rate of training data is low. In scenario 2, the size of training set (621 samples) holds constant and number of testing samples gradually increases from 62 to 5589. Model precisions evaluated at the 9 ratios are not significantly different for both methods, which are about 0.63, 0.74, and 0.45 for DTT, PH, and EW on average, respectively (Fig. 4b). In scenario three, the ratio of 9:1 (training vs. testing) holds constant, and the population size gradually decreases from 6210 to 690. Along with the decrease of population size, precisions of both methods gradually drop starting from about 0.75, 0.79, and 0.65 for DTT, PH, and EW, respectively; but precisions of the two methods are equivalent to each other (Fig. 4c). In scenario 4, the ratio of 1:9 (training vs. testing) holds constant, and the population size gradually decreases from 6210 to 690. Along with the decrease of population size, precisions of both methods gradually drop starting from about 0.63, 0.72, and 0.48 for DTT, PH, and EW, respectively; but the overall precision of LightGBM is better than that of rrBLUP (Fig. 4d).

The above analyses collectively indicate the importance of sampling rate of training data in determining the baseline precision of GS prediction, which is a critical factor needed to consider when designing a GS project. When the size of training set is fixed at 621 samples accounting for 10% of the population, the baseline precisions for DTT, PH, and EW are 0.637, 0.716, and 0.467 averaged from the results of LightGBM prediction in the four scenarios; in contrast, the baseline precisions for the three traits are 0.623 (DTT), 0.706 (PH), and 0.503 (EW) for rrBLUP (Additional file 1: Table S2). We may also conclude that LightGBM may outperform rrBLUP, when the sampling rate is lower than 10% and the size of training samples is much smaller than that of testing samples.

### LightGBM performs classification prediction

**Fig. 5:**
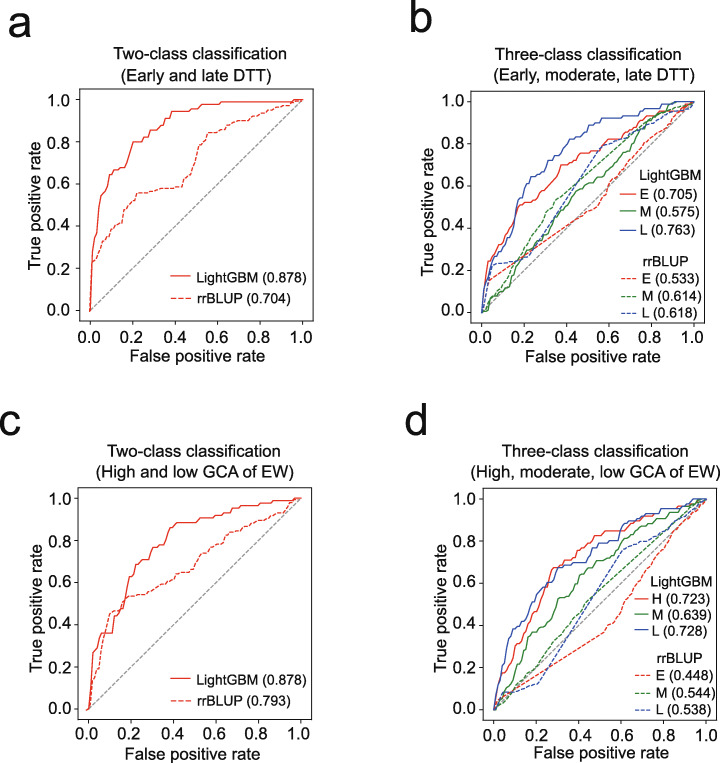
Performance of classification prediction by LightGBM and rrBLUP. **a** Comparison on classifying Zheng58 F_1_s with early and late DTT by LightGBM and rrBLUP. The numbers in the brackets are the AUC (area under the curve) values to indicate model precisions of the two methods. **b** Comparison on classifying Zheng58 F_1_s with early, moderate, and late DTT. **c** Comparison on classifying maternal lines with high and low GCA of EW. **d** Comparison on classifying maternal lines with high, moderate, and low GCA of EW. The computation of the general combining ability (GCA) value for each maternal line is based on the EWs of Zheng58 and Jing724 F_1_s

LightGBM can also perform classification prediction with binary or multi-class labels suitable for qualitative traits. To test LightGBM for classification tasks, we categorized the 1428 Zheng58 F_1_s into three classes of samples with early (top 25% DTT), moderate (25 to 75% DTT), and late (lowest 25% DTT) flowering times. The two-class classification with fivefold CVs showed excellent discrimination of early and late-flowering F_1_s, as reflected by a high area under the curve (AUC) value of 0.878 (Fig. 5a and Additional file 1: Fig. S6a). In contrast, the AUC of discriminating early- and late-flowering F_1_s by rrBLUP was 0.704. As for the three-class classification by LightGBM, the precisions for discriminating early, moderate, and late-flowering F_1_s were 0.705, 0.575, and 0.763, respectively; while by rrBLUP, AUCs for the three corresponding classes were 0.533, 0.614, and 0.618 (Fig. 5b and Additional file 1: Fig. S6a).

In breeding practice, breeders typically make decisions via binary judgement, even if a trait is quantitative. For instance, in test-crossing experiments, breeders typically select the top 15 to 25% lines based on the general combining ability (GCA) of the grain yield computed on the basis of the average performance of their F_1_s crossed with two or three testers. To test whether LightGBM can assist line selection, the 1428 CUBIC lines were categorized into three sample classes with high (top 25%), moderate (25 to 75%), and low (lowest 25%) GCAs of EW computed based on the F_1_s crossed with Zheng58 and Jing724 (Methods). Subsequently, two-class and three-class classifications were performed to evaluate the model precision. While the model achieved a high precision with AUC = 0.793 for the two-class classification, the precisions for discriminating the samples with high, moderate, and low GCAs were 0.723, 0.639, and 0.728, respectively (Fig. 5c, d and Additional file 1: Fig. S6b). In contrast, AUC for two-class classification by rrBLUP was 0.678, and AUCs for classifying high, moderate, and low GCAs by rrBLUP were 0.448, 0.544, and 0.538, respectively. These results confirm the effectiveness of LightGBM in performing classification prediction that can facilitate decision-making by binary judgement for breeders.

### Interpretation of highly effective SNPs in biology

**Fig. 6:**
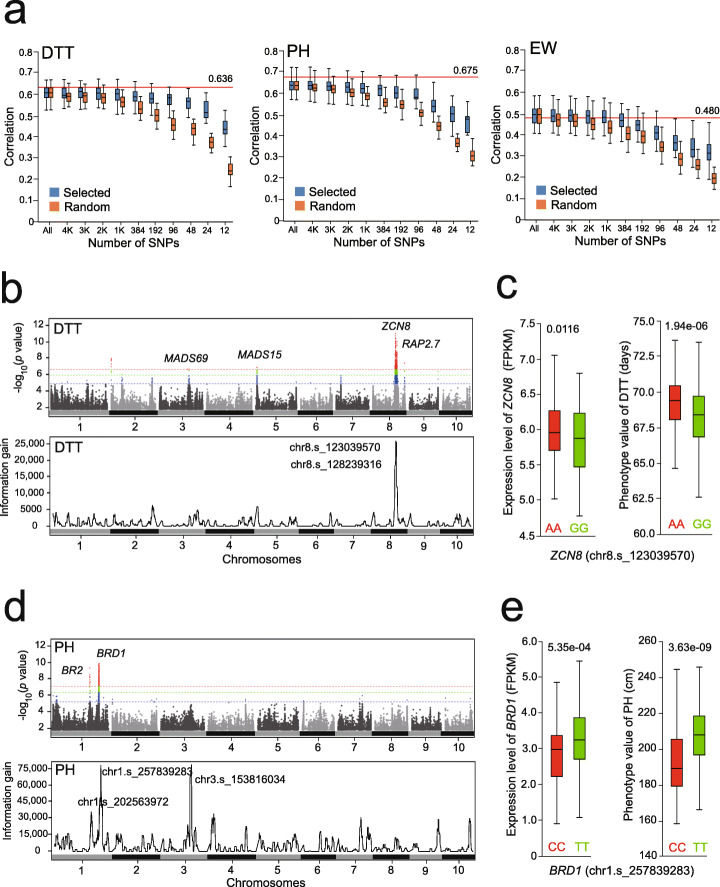
Interpretation of highly effective SNPs recognized by LightGBM. **a** Ten grades of SNPs selected based on ranked IGs from high to low are used as features to test the precisions of LightGBM on DTT, PH, and EW, in parallel comparison with the use of 32,559 SNPs (All) and the same number of SNPs randomly selected from the genome. For each grade of the SNP number, fivefold CVs are performed to generate a distribution of the correlations. The red horizontal lines represent the precisions of rrBLUP on DTT, PH, and EW when using all the 32,559 SNPs for prediction. **b** Comparison of the IG distribution of the selected 384 highly effective SNPs with the GWAS signals on DTT across the 1428 maternal lines. **c** The 411 maternal lines are divided into AA and GG subgroups based on the genotype of the top SNP chr8.s_123039570 in the QTL of *ZCN8*. The two subgroups of lines exhibit significantly different expressions of the associated gene *ZCN8* (*p* = 0.0116, *t*-test) and DTT phenotype (*p* = 1.94e−06, *t*-test). **d** Comparison of the IG distribution of the selected 384 highly effective SNPs with the GWAS signals on PH across the 1428 maternal lines. **e** The 411 maternal lines are divided into CC and TT subgroups based on the genotype of the top SNP chr1.s_257839283 in the QTL of *BRD1*. The two subgroups of lines exhibit significantly different expressions of the associated gene *BRD1* (*p* = 5.35e−04, *t*-test) and PH phenotype (*p* = 3.63e−09, *t*-test)

During model training, LightGBM infers the predictive effectiveness for each SNP by computing the information gain (IG) value to represent feature importance. The higher the IG, the more effective the genotype of an SNP in discriminating the phenotypes. To validate the effectiveness of the features recognized by LightGBM, ten sets of features, including the top 12 to 4000 SNPs sorted by IGs generated from model training on 1428 maternal lines, were compiled (Methods). Simultaneously, the same number of SNPs for each feature set were randomly selected from the genome as controls. With the decreased numbers of LightGBM-selected SNP from 32,559 to 96, precisions for the three traits gradually dropped without great difference, compared to the results when using genome-wide SNPs (Fig. 6a). In contrast, the precisions dropped more drastically with the decrease in the number of randomly selected SNPs. Hence, the feature importance analysis by LightGBM is a practical utility to generate condensed marker panels (e.g., 96 to 384 markers) for a designated pool of breeding materials, enabling the use of a sample-multiplexing solution, such as the GBTS (genotyping by targeted sequencing) platform [29], to lower the genotyping expense for large breeding populations.

Presumably, the decision trees generated by LightGBM from the boosting iterations may accurately capture the trait-associated SNPs that significantly contribute to phenotype variations. To test our assumption, we compared the genomic distribution of the IGs of the SNPs with the GWAS signals detected across the 1428 maternal lines (Methods). As expected, the overall IG distribution was in accordance with the GWAS signals (Fig. 6b). The top two SNPs, chr8.s_123039570 and chr8.s_128239316, fell into the QTLs of *ZCN8* and *RAP2.7*, respectively, which form the previously reported *RAP2.7*–*ZCN8* flowering-time regulatory module [30, 31]. Therefore, the SNP chr8.s_123039570 is perhaps associated with genomic variations that may influence *ZCN8* expression, as it encodes the florigen in maize to directly initiate floral transition. To test whether the genotype (AA and GG) of chr8.s_123039570 correlates with *ZCN8* expression, we examined the expression levels of *ZCN8* in 412 CUBIC lines divided into *ZCN8*-AA (268 lines) and *ZCN8*-GG (144 lines) groups. As expected, both the DTT phenotypes and *ZCN8* expression concordantly exhibited a significant difference between the two groups (Fig. 6c). Subsequently, we compared the IG distribution and GWAS signals of PH and found that the top first (chr1.s_257839283) and second (chr3.s_153816034) SNPs fell into the QTLs of *BRD1* (*Brassinosteroid C-6 oxidase 1*) and *MADS69*, respectively, and a moderate SNP (chr1.s_202563972) fell into the *BR2* (*Brachytic2*) QTL (Fig. 6d). While *BRD1* and *BR2* have been functionally characterized in regulating plant height, *MADS69* is a MADS-box transcription factor functioning upstream of the *RAP2.7*–*ZCN8* module to regulate the flowering time [32–34]. To test the correlation between chr1.s_257839283 (CC and TT), PH phenotypes, and *BRD1* expression, the 412 CUBIC lines were divided into groups of *BRD1*-CC and *BRD1*-TT containing 96 and 316 lines, respectively. Similar to the result obtained for DTT, both the PH phenotypes and *BRD1* expression were significantly different between the two subgroups (Fig. 6e).

Unexpectedly, the SNP chr3.s_153816034 associated with the QTL of *MADS69* was recognized by LightGBM on PH, which were moderately detected by GWAS of DTT but undetected by GWAS of PH. *MADS69* promotes flowering by activating the florigen *ZCN8*; however, its recognition by LightGBM on PH is worthy of further investigation. Based on the genotype of chr3.s_153816034 (CC and TT), the CUBIC lines were divided into *MADS69*-CC (124 lines) and *MADS69*-TT (317 lines) groups, in which the expression of *MADS69* between the two groups exhibited a significant difference (*p* = 0.017). Correspondingly, the *MADS69*-TT group with a higher expression of *MADS69* showed phenotypes of significantly shorter PH and earlier DTT than the *MADS69*-CC group (Additional file 1: Fig. S7a). Therefore, there could be a possible interaction between *BRD1* and the *MADS69*-*ZCN8* regulatory module that coordinates the balance between PH and DTT; this was probably overlooked by GWAS. As the 1428 CUBIC lines were derived from the 24 founder lines, we examined the expression levels of *BRD1*, *MADS69*, and *ZCN8* in correlation with the DTT and PH phenotypes in the leaf tissues in the maize V1 developmental stage. Among the 23 lines with homozygous alleles, 18 lines bearing *BRD1*-TT showed lower expression of *BRD1* but higher expressions of *ZCN8* and *MADS69*, compared with the 5 lines bearing *BRD1*-CC, which were in concordance with the phenotypes of the 18 lines showing shorter PH and earlier DTT (Additional file 1: Fig. S7b). This correlation is consistent with the fact that a high expression of *MADS69* may promote early DTT and cause short PH as a result of the early termination of vegetative growth. In summary, these analyses collectively indicate that the highly effective SNPs recognized by LightGBM are mostly trait-associated markers, somehow related to genomic variations responsible for regulating the causal genes and related phenotypes.

### LightGBM facilitates gene mining by enhancing GWAS sensitivity

**Fig. 7:**
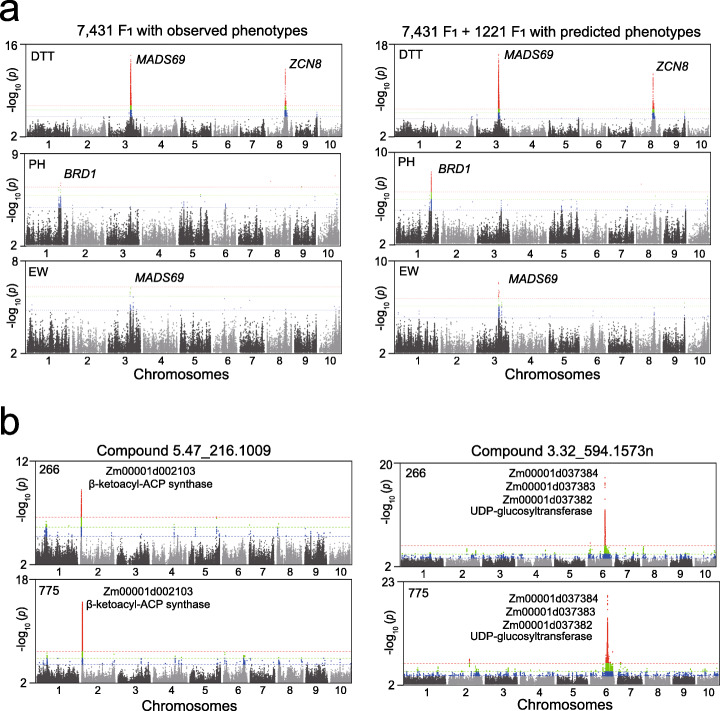
Enhancement in GWAS sensitivity by LightGBM prediction. **a** GWAS signals on DTT, PH, and EW were enhanced after adding the 1221 F_1_s with predicted phenotypes to the 7431 training samples with observed phenotypes. The blue, green, and red dashed lines represent the *p* values of 1e−5, 1e−6, and 2.2e−7, respectively. **b** GWAS signals on metabolic compounds of 5.47_216.1009 (left panel) and 3.32_594.1573n (right panel) were enhanced after adding the 509 lines with predicted phenotypes to the 266 training samples. The blue, green, and red dashed lines represent the *p* values of 1e−5, 1e−6, and 2.2e−7, respectively

GWAS is an efficient way to identify trait-associated genes and understand the genetic architecture underlying a trait. With the rapid advancement of next-generation sequencing, genotyping expense has been significantly reduced, while phenotyping has become the bottleneck for GWAS in the case of large-scale populations. For quantitative traits controlled by multiple genes, the population size is particularly critical for GWAS detection power [35]. Presumably, if genomic prediction is precise, the predicted phenotypes may be used for unmeasured samples to carry out a GWAS analysis. Thus, phenotyping expense can be saved; however, an ideal population size should be ensured. To test this assumption, the phenotypes of DTT, PH, and EW of 1221 Zheng58 F_1_s were predicted by LightGBM using the 7431 samples (6210 F_1_s + 1221 Jing724 F_1_s) as training set. Then, we compared the GWAS signals derived from the 7431 samples with observed phenotypes and the 8652 samples containing the 1221 F_1_s with predicted phenotypes. As for the DTT trait, both *MADS69* and *ZCN8* peaks were detected in the two populations, and the 8652 samples exhibited enhanced SNP effects, likely due to the augmentation of population size (Fig. 7a, upper panels). Similarly, GWAS of PH and EW also exhibited enhanced signals associated with the *BRD1* and *MADS69* genes (Fig. 7a, middle and lower panels).

Furthermore, we tested the predictability of LightGBM on the contents of metabolic compounds that may be considered a type of qualitative trait determined by few biosynthetic enzymes. The contents of the two uncharacterized metabolites (5.47_216.1009 and 3.32_594.1573n) measured in a core germplasm collection of 266 lines [36] were used as the phenotypes to predict their contents in another 509 unmeasured lines. Subsequently, the GWAS of the two metabolites was performed individually in the 266 lines and 775 (509 + 266) lines. As for 5.47_216.1009, one significant peak was detected in the 266 lines with the measured contents, corresponding to a candidate gene Zm00001d002103 encoding β-ketoacyl-ACP synthase involved in the biosynthesis of fatty acid based on CornCyc annotation (Fig. 7b, left panel). In the 755 lines including the 509 samples with the predicted contents, the same peak associated with gene Zm00001d002103 was found with enhanced signals. The GWAS of the metabolite 3.32_594.1573n in the 266 lines detected a cluster of tandemly duplicated genes encoding UDP-glucosyltransferases (Zm*UGTs*), showing the same GWAS signals in the 775 lines containing predicted samples (Fig. 7b, right panel). The three Zm*UGTs* have been previously detected by metabolome-GWAS in maize, revealing their involvement in flavonoid biosynthesis [37] (Additional file 1: Fig. S8). Therefore, GWAS identification of the genes originally detected in the training population not only validates the precision of LightGBM, but also demonstrates the utility of the GS strategy for gene mining on unexploited germplasm with significantly reduced phenotyping expense. However, it should be noted that this strategy may be only applicable to the trait with high predictability, and the QTL effect might not be accurately estimated using predicted phenotype. Thus, this approach is only suitable for a rough mapping of trait-associated QTLs on unexploited germplasm to identify genomic regions sharing similar haplotypic patterns between the training and predicting populations.

### Implementation of LightGBM to facilitate genomic breeding in crops

**Fig. 8:**
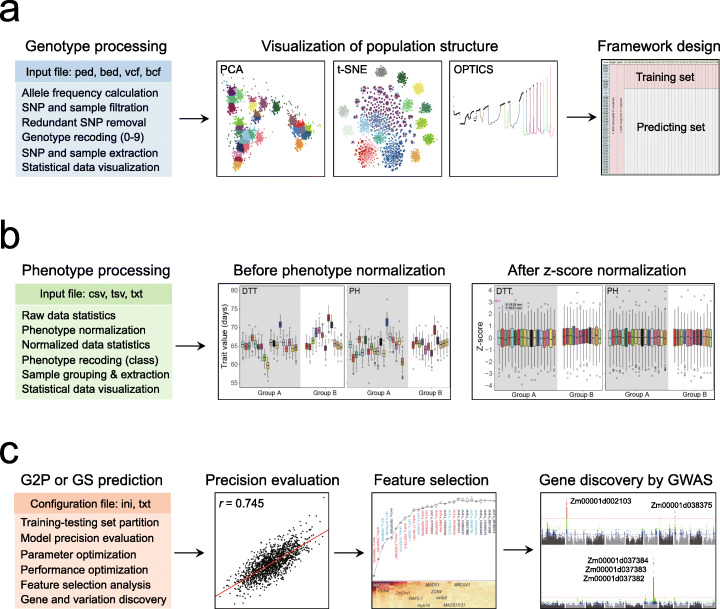
CropGBM offers streamlined analysis to assist genomic design breeding. **a** Genotype analytical module includes genotypic data analysis pipeline, visualization of population structure, and OPTICS analysis to facilitate the design of an optimal predictive framework. **b** Phenotype analytical module includes basic statistics of phenotypic data and normalization function. **c** G2P prediction module utilizes LightGBM as the kernel algorithm to construct a predictive model and perform related ML analysis

LightGBM is among the most popular ML algorithms for Big Data analytics in the industry, owing to its ultra-high efficiency in handling structured data with high feature dimensions and large sample sizes [23]. In the seed industry, a breeding program may last for years, accumulating tremendous amount of genotype and phenotype data that allow implementing data-driven decision-making to facilitate crop breeding. Our analysis showcased the capability of ensemble learning in GS-assisted breeding. Therefore, we utilized LightGBM as the kernel algorithm to develop a toolbox, namely CropGBM, for streamlined GS prediction to facilitate genomically designed crop breeding. CropGBM includes three main analytical modules covering the steps of genotype analysis, phenotype analysis, and GS prediction. The first module performs a series of processing steps on genotype data and converts genotypic characters to numeric features (Fig. 8a). Subsequently, the sample population structure is analyzed using PCA, t-SNE, and OPTICS (Ordering Points to identify the clustering structure) algorithms (Methods). While the PCA and t-SNE are commonly used methods for visualizing the population structure, the utility of OPTICS is that it can quantitatively evaluate the genetic distance within and between subpopulations and perform nonlinear clustering of samples (Fig. 1b). The results of OPTICS may facilitate the design of an optimal predictive framework by appropriately partitioning the samples into training, testing, and predicting sets, which can help maximally avoid model overfitting due to the influence of population stratification. The second module performs basic statistics and data visualization on the phenotypes of the samples (Fig. 8b). If population stratification occurs in the phenotype between subpopulations, *z*-score normalization within each subpopulation is highly recommended. The third module performs GS prediction for either regression or classification analysis, supported by hyperparameter tuning using grid search for model optimization (Fig. 8c). During model training, a feature selection is performed to recognize highly effective SNPs for designing highly condensed marker panels.

CropGBM supports acceleration by multi-threading and GPU computing. As the feature number and sample size influence the performance of LightGBM, we tested its computing time and memory consumption with different magnitudes of simulated data while comparing it with rrBLUP. The benchmark testing showed that the performance of rrBLUP is restricted by the sample size but insensitive to the SNP number. Training rrBLUP on the 50,000 samples with 10,000 SNPs took over 17 h and 116 GB of memory, whereas LightGBM required only 8 min and 20 GB of memory on the same server (Additional file 1: Table S3). On 100,000 samples, rrBLUP failed to train the model, but LightGBM completed the training in 15 min with 40 GB of memory. If GPU acceleration was enabled, the training time was shortened to only 4 min on 100,000 samples. We also tested BLUPF90, a widely used free GS tool in animal breeding, whose speed performance has been maximized in exchange of increased memory usage [38]. The benchmark test was performed on 25,000 samples on the same sever for testing rrBLUP and LightGBM, as this number of genotyped samples is the upper limit of population size that BLUPF90 can take. BLUPF90 spent 45 min and consumed 36 GB memory to accomplish model training. Therefore, LightGBM features superior capability of exploiting extremely large datasets with an ultra-high efficiency.

## Discussion

Accelerated crop breeding refers to the integration of multifaceted technologies, including doubled haploid (DH) techniques, speed breeding (SB), genomic selection (GS), and genome editing [2–5]. Owing to the fast advance of DH and SB techniques, a seed company may produce tens of thousands inbred lines per year. With greatly reduced genotyping expense, genotype-based screening of superior lines by GS model has become an essential component in modern breeding programs for major crops like maize, rice, wheat, and soybeans. As a breeding program may last for multiple years, the magnitude of training data, including genotypes and phenotypes of F_1_ hybrids and inbred lines, may be considerably high. Most popular GS tools, such as rrBLUP, Sommer, BLUPF90, and ASReml, employ mixed linear model (MLM) for prediction and consume a considerable amount of computing resource to solve the model [10, 38, 39]. Therefore, ultra-efficient ML techniques have been desired to facilitate data-driven decision-making in crop breeding. To this end, we assessed multiple ML algorithms in this work and determined LightGBM as the optimal solution for GS in terms of both computing efficiency and prediction precision. On hundreds of thousands of samples, LightGBM can complete model training in minutes on a desktop server, particularly when GPU acceleration is enabled. The excellent performance of LightGBM might be algorithmically attributed to its main difference in tree construction from other GB algorithms like GBDT, XGBoost, and CatBoost. LightGBM adopts the strategy of leaf-wise tree growth which identifies the “best” leaf with the highest gain and only splits the best leaf, resulting in an asymmetrical tree. In contrast, other GB variants grow a tree level-wise, meaning that each node at the same level is split to child nodes. A tree structure built by leaf-wise growth may better reflect genotypic interactions between biologically related genes than a tree by level-wise growth. This may explain its higher precision of LightGBM than other GB variants. For example, the genotype (AA and GG) of Gene-X separates the total samples into AA and GG branches exhibiting phenotypic variations; the samples in the AA branches are further separated by the genotype of (TT and CC) of Gene-Y, while no separation occurs on the GG branch. The likely biological principle underlying this scenario is that Gene-Y may execute a stronger function under the AA background of Gene-X rather than under the GG background. Thus, a putative interaction between Genes X and Y may exist, probably explained by a nonlinear epistasis effect. Subsequently, if the population is sufficiently large, genome-wide epistatic interactions might be accumulatively learned by LightGBM. The resulting tree presumably resembles to a network of causative genes determining a trait.

Because of the abovementioned merits of LightGBM, we then implement it as a one-stop toolbox CropGBM. In addition to the core GS function, it also integrates multiple novel features and analytical functions, such as genotype and phenotype data preprocessing and recoding, dimensionality reduction and population visualization via PCA and t-SNE algorithms, sample classification via K-means and OPTICS clustering, feature selection via information gain (IG) analysis to assist marker design, GPU-enabled acceleration, and so on (Additional file 1: Table S4). Inclusion of diverse utilities in CropGBM is to ensure the best performance of GS prediction, considering the complex scenarios in crop hybrid breeding.

Our work followed a standard procedure whereby an ML system is built and included a series of assessments on the factors influencing the precision and stability of a predictive model. These assessments are critically important and highly recommended to perform prior to carry out a GS task in crop breeding, as model performance may vary by different species, populations, and traits [6]. Using a compiled dataset of genotypes and phenotypes in six plant species [6], we compared LightGBM with the other 11 GS tools on predicting the trait of plant Height (HT) (Additional file 1: Fig. S9a). LightGBM ranked as the top one algorithm in Rice and Soy, but its precision (*r* = 0.40) was lower than that of rrBLUP (*r* = 0.44) in maize. One possible reason is that the 391 inbred lines in the core germplasm represent a wide range of genetic diversity as the panel contains temperate maize, tropical maize, popcorn maize, sweet maize in America, and some exotic maize [40]. rrBLUP adopts maximum-likelihood algorithm to estimate fixed effects and SNP effects that are used to predict phenotypes from genome-wide SNPs. In contrast, LightGBM scans the total feature set to select a small number of highly effective SNPs to perform prediction. If the complexity of the population is too high, the limited number of SNPs selected by LightGBM may not sufficiently quantify all the genetic variations contributing to the trait, thus lowering the prediction precision. Therefore, LightGBM is more effective on breeding population without severe population stratification, such as the paternal half-sibling population as we used in this study. In actual practice of employing GS in crop breeding, breeders also have to consider the cost spent on phenotyping training samples which account for a small portion (usually 10 to 15%) of the total samples. Thus, it is highly necessary to estimate the correlation between prediction precision and sampling rate in advance, so that precision and cost may be optimally balanced. In addition, feature dimension is also an important factor to consider when building a ML system, as excessive SNP features may not only cause unnecessary computing cost but also lead to dimension explosion for certain ML algorithms. Selection of the optimal number of SNPs also depends on the genetic diversity of a population. For instance, the germplasm panel with broad genetic diversity requires more features (~ 20,000 SNPs) than the CUBIC population (~ 3000 SNPs) to achieve an acceptable precision (Fig. 6a and Additional file 1: Fig. S9b).

## Conclusion

Various speed breeding techniques have accelerated the production of inbred lines in major crops like maize, rice, wheat, and soybeans, enforcing the employment of genomic prediction techniques, such as the genomic selection (GS) model, to assist selection of superior lines or hybrids. LightGBM is an ensemble learning framework, which adopts the strategy of leaf-wise tree growth to construct decision trees and features ultrafast efficiency in coping with large dataset. We implemented LightGBM as a one-stop toolbox CropGBM to assist the employment of GS in crop breeding. CropGBM exhibits superior performance in coping with large sample sizes, which may help accomplish model training on hundreds of thousands of samples in a matter of minutes. It also includes novel features and analytical modules to cover multiple aspects of genotype and phenotype data analysis, population genetic analysis, feature importance analysis, and GS prediction. These functions may ensure the best performance of GS prediction, considering the complex scenarios in crop hybrid breeding. We also proved that the intrinsic mechanism of ensemble learning in GS prediction is interpretable in biology, although an ML model has been referred to as a black box. This is based on the results showing that the highly effective SNPs selected by LightGBM exhibit significantly correlated divergence in the genotypes, phenotypes, and expressions of trait-associated genes. Thus, these correlations can form a theoretical basis for implementing data-driven genomic design for accelerated breeding in crop species.

## Methods

### Genotype and phenotype data

Liu et al. previously described the development of the maternal CUBIC pool including the 1428 inbred lines crossed from the 24 founder females [27]. A total of 4,549,828 high-quality SNPs called from the whole-genome resequencing of the 1428 CUBIC lines and 30 paternal testers were used for the GWAS analysis in Fig. 7. The procedure of SNP calling and genotype processing of the 1458 parental lines has been described by Liu et al. [27]. To reduce the feature dimensions, the 32,559 SNPs used for the GS and ML prediction were selected on the basis of the common SNPs between the 4.5 million SNPs and the 65,620 SNPs used in a 65 K maize SNP chip that has been widely employed for genomic breeding in maize. The genotypes of the 42,840 F_1_ hybrids were inferred by combining the maternal and paternal alleles derived from the 1428 and 30 lines, respectively. The three phenotypic traits, namely the days to tasseling (DTT), plant height (PH), and ear weight (EW), of the 1458 maternal lines and the 8652 F_1_ hybrids were measured in five locations. Details of collecting phenotypes were described in Xiao et al. [26]. To reduce the influence of the environment, the best linear unbiased prediction (BLUP) value of each F_1_ hybrid and each parental line was computed for the phenotypic data in the five locations over 2 years, using the mixed linear model in the R package “lme4.” The BLUP values for each phenotype were then used for the subsequent analysis.

### Procedure to build the ML system for GS prediction

The procedure of constructing an ML system for a specific task involves multiple steps to ensure an objective assessment of the system. For model selection, five basal methods, including SVR, ANN, KNN, RF, and GB, were compared with rrBLUP in parallel. For the feature selection, 32,559 SNPs were selected from the original set of 4.5 million SNPs based on professional knowledge in maize population genetics. For the model evaluation, we designed multiple predictive frameworks and different ratios of training and testing samples to test the model precision and model stability. When ensemble learning is determined as the optimal approach, three more variants of the GB method, namely XGBoost, CatBoost, and LightGBM algorithms, were tested in parallel with rrBLUP. To further optimize the LightGBM model, additional functions, including feature format conversion, hyperparameter grid search, classification prediction, and GPU acceleration, were implemented. The feature importance analysis was based on computing a score of the information gain to represent the predictive effectiveness for each SNP. To interpret the underlying mechanism of LightGBM in G2P prediction, the 384 highly effective SNPs recognized by LightGBM were compared with the trait-associated QTLs identified by the GWAS analysis to prove their regulatory association with gene expression and phenotype variations. Finally, a parallel benchmark testing was performed on the same server to test the computing performance of CropGBM and rrBLUP. The server was equipped with a Xeon E5-2665 CPU clocked at 2.40 GHz (8 cores × 2), 128 GB of memory, and an NVIDIA GeForce GTX-P8 GPU (1080 × 4)

### Evaluation of model precision

We used different CV methods to evaluate the model precision based on different predictive goals and partitions of the training and testing samples. Significance level (*p* value) of statistical test for the comparison of rrBLUP and LightGBM used in the corresponding figures is presented in Additional file 1: Table S5.

1. To evaluate the five basal ML models using the 6210 (207 maternal × 30 paternal lines) F_1_s (Fig. 1c), 29 F_1_ populations were used as the training samples to predict the rest of the F_1_ population. This procedure was repeated 30 times to test the precision across all the 30 F_1_ populations, so that a distribution of the precision (*r*, Pearson’s correlation between the predicted and measured phenotypes) can be generated for each model.
2. To evaluate the influence of the sampling rate of training data on prediction precision, four scenarios were designed using the population of 6210 F_1_s, namely fixed size of testing set (621 samples), fixed size of training set (621 samples), fixed ratio of 9:1 (training vs. testing), and fixed ratio of 1:9 (training vs. testing) (Fig. 4). For the first two scenarios, 9 ratios (9:1, 7:1, 5:1, 3:1, 1:1, 1:3, 1:5, 1:7, and 1:9) of training and testing were set, and then the training and testing sets were randomly built for 30 times at each ratio to generate a precision distribution for LightGBM and rrBLUP. For the last two scenarios, 9 population sizes with fixed ratio of 9:1 and 1:9 were set, and then the training and testing sets were randomly built for 30 times at each size.
3. To evaluate the influence of parental compositions on the prediction (Fig. 2b), the 6210 F_1_s were partitioned into 30 subgroups, 25 of which were used as training samples to predict the F_1_s in the rest of the 5 subgroups. This procedure was repeated 30 times to generate a distribution of the precision.
4. To evaluate the precision between different F_1_ populations (Fig. 2c), the 1428 Zheng58 F_1_s were first used as training samples to predict the 1428 Jing724 F_1_s; then, the 1428 Jing724 F_1_s were first used as training samples to predict the 1428 Zheng58 F_1_s.
5. To evaluate the model fitting ability on different GB variants (Fig. 3a), the 8652 F_1_s with measured phenotypes were used to first train the models and then generate the predicted phenotypes for the 8652 F_1_s. Subsequently, Pearson’s correlations between the predicted and measured phenotypes for the 8652 F_1_s were computed to represent the fitting ability of each model.
6. To compare the precision between LightGBM and rrBLUP with and without parental phenotypes as additional features (Fig. 3c), 6210 F_1_s + 1221 Zheng58 F_1_s were first used as training samples to predict the 1221 Jing724 F_1_s; then, 6210 F_1_s + Jing724 F_1_s were first used as training samples to predict the 1221 Zheng58 F_1_s.
7. To test the classification task performed by LightGBM (Fig. 5), the 1428 maternal lines were labeled with early, moderate, and late DTT (GCA of EW), and fivefold CVs were performed to train the model, followed by generating the ROC curve and an AUC value to represent the precision. To test the classification task by rrBLUP (Additional file 1: Fig. S6), the 1428 maternal lines were first labeled with early, moderate, and late DTT (GCA of EW) according to observed phenotypes. After rrBLUP prediction, the lines were relabeled according to the values of predicted phenotypes, followed by generating the ROC curve and an AUC value to represent the precision.
8. To evaluate the effectiveness of feature selection by LightGBM (Fig. 6a), sampling of 80% of the 1428 maternal lines were repeated for 30 times, and IGs were computed at each time of repeat to select highly effective SNPs to retrain LightGBM, followed by predicting phenotypes of the rest 20% lines. Detailed procedure was described in the section below.

### Feature importance analysis with information gain (IG)

Entropy is a mathematical indicator reflecting the degree of dispersion of a group of samples. A low entropy value indicates a low degree of sample dispersion. As the decision tree takes the average value of the phenotypes in the leaf nodes as the predicted value, the lower the entropy in the leaf node of the training samples, the higher the precision of the predicted value. In the feature importance analysis by LightGBM, the information gain (IG) value is used to represent the change in the entropy before and after separating the samples, which may reflect the effectiveness of a tree node (SNP) in terms of discriminating two branches of samples that exhibit a strong phenotypic difference. Therefore, the SNPs with high IGs indicate a high power in classifying samples. After completing all the iterations of tree building during model training, the IG values for each SNP are summed to represent its feature importance that may reflect its power on association between the genotype and phenotype. To exclude the influence of SNP effects from testing samples, the IGs were first computed by LightGBM in each time of sampling of 80% lines as training set, and the top 12, 24, 48, 96, 192, 384, 1000, 2000, 3000, and 4000 SNPs were selected, which were based on ranking the summed IGs from high to low, as features to retrain LightGBM. The model was then used to predict phenotypes of the rest 20% lines. The sampling was repeated for 30 times to generate 30 Pearson correlations used to plot precision distribution for each set of SNPs. Meanwhile, the same procedure was applied on the same number of SNPs randomly sampled from the 32,559 SNPs to plot precision distribution as the contrast. At last, rrBLUP was performed on the 1428 maternal lines using 32,559 SNPs to compute baseline precisions for the three traits.

### Conversion of genotypic characters to numeric features

For both rrBLUP and machine learning tools, the genotypic characters should be first converted to numeric features based on the allele frequency of each SNP in the training and testing populations. When using the 0, 1, 2 coding scheme, the homozygous genotype (AA) of the two major alleles is coded as 0, heterozygous genotype (AB) of one major and one minor allele is coded as 1, and the homozygous genotype (BB) of the two minor alleles is coded as 2. In addition to the 0, 1, 2 coding scheme, LightGBM may employ an alternative 0–9 coding scheme to represent all the forms of genotypes, such as the converting rule used in CropGBM, as follows: AA (0), AT (1), TA (1), AC (2), CA (2), AG (3), GA (3), TT (4), TC (5), CT (5), TG (6), GT (6), CC (7), CG (8), GC (8), GG (9).

### Calculation of general combining ability (GCA)

The general combining ability (GCA) is the average value of the inbred line based on its behavior in crosses with other lines. It is calculated as follows:

$$ {GCA}_i=\overline{X_i}-\overline{X} $$

where *GCA*_*i*_ represents the GCA of parent *i*, $\overline{X_i}$ is the average value of the *i* parental hybrid offspring, and $\overline{X}$ is the population mean of all hybrid offspring.

### Versions of tools and packages

Machine learning tools in Python sklearn package (version, https://scikit-learn.org/stable/);

Support vector regression (SVR, SVR);

Random forests (RF, RandomForestRegressor);

Artificial neural network (MLP, MLPRegressor);

K-nearest neighbor (KNN, KNeighborsRegressor);

Gradient boosting regression tree (GB, GradientBoostingRegressor);

PCA analysis (decomposition);

t-SNE analysis (manifold);

OPTICS analysis (cluster);

eXtremeGB (XGB, XGBoostRegressor);

Categorical GB (CatGB, CatBoostRegressor);

BLUP (R package ‘lme4’);

LightGBM (version 2.2.4, https:// lightgbm.readthedocs.io/);

Genomic selection analysis: rrBLUP (version 4.6, https://cran.r-project.org/src/contrib/Archive/rrBLUP/rrBLUP_4.6.tar.gz);

GWAS analysis: GEMMA (version 0.97, https://github.com/genetics-statistics/GEMMA/tree/gemma-0.97-preview);

Manhattan plot: CMplot (https://cran.r-project.org/web/packages/CMplot/index.html)

Metabolic pathway: CornCyc (https://corncyc-b73-v4.maizegdb.org)

PLINK (version 1.90, https://www.cog-genomics.org/plink/)

Evolution analysis: MEGA (version 7.0, https://www.megasoftware.net/)

Cladogram: EvolView (https://www.evolgenius.info/evolview/)

Other scripts for CropGBM (https://github.com/YuetongXU/Cropgbm-Paper)

## Supplementary Information

**Additional file 1.** Supplemental Tables S1 to S5 and Figures S1 to S9.

**Additional file 2.** Review history.

## Data Availability

Source codes and the tutorials of CropGBM toolbox are academically free at GitHub: https://ibreeding.github.io/. Codes and dataset used for the analysis in this article are available at GitHub: https://github.com/YuetongXU/Cropgbm-genomebiology [41], and the public repository Zenodo.org at DOI: 10.5281/zenodo.5431934 [42].
