# Supplementary material for: LightGBM: accelerated genomically designed crop breeding through ensemble learning
Source: Genome Biol. 2021 Sep 20;22:271. doi: 10.1186/s13059-021-02492-y (PMC8451137; doi:10.1186/s13059-021-02492-y)
Supplement: Supplementary file 1 — Additional file 1. Supplemental Tables S1 to S5 and Figures S1 to S9. [file 13059_2021_2492_MOESM1_ESM.docx]

**Supplemental tables and figures for the Manuscript**

**LightGBM: accelerated genomically-designed crop breeding through ensemble learning**

Jun Yan^1*^, Yuetong Xu^1*^, Qian Cheng^2^, Shuqin Jiang^1^, Qian Wang^1^, Yingjie Xiao^3^, Chuang Ma^2^, Jianbing Yan^3#^ and Xiangfeng Wang^1#^

^1^ National Maize Improvement Center, Department of Crop Genomics and Bioinformatics, College of Agronomy and Biotechnology, China Agricultural University, Beijing 100193, China

^2^ Key Laboratory of Biology and Genetics Improvement of Maize in Arid Area of Northwest Region, Ministry of Agriculture, Northwest A&F University, Shaanxi, China

^3^ National Key Laboratory of Crop Genetic Improvement, Huazhong Agricultural University, Wuhan 430070, China

^*^ These authors contribute equally to this work

^#^ Correspondence: Xiangfeng Wang ([xwang@cau.edu.cn)](mailto:xwang@cau.edu.cn)) and Jianbing Yan ([yjianbing@mail.hzau.edu.cn)](mailto:yjianbing@mail.hzau.edu.cn))

**Supplemental Table. 1.** Best hyperparameter values of the six machine learning models selected by grid search.

| **Model** | **Function in sklearn** | **Hyperparameter** | **Values tested by grid search** | **Best Value** |
| --- | --- | --- | --- | --- |
| GB | sklearn.ensemble.GradientBoostingRegressor() | learning_rate | 0.01, 0.05, 0.1, 0.2, 0.5 | 0.1 |
|  |  | n_estimators | 10, 50, 100, 500, 1000 | 100 |
|  |  | min_samples_split | 2, 10, 20, 40 | 2 |
|  |  | min_samples_leaf | 1, 5, 10, 20 | 1 |
|  |  | max_depth | 3, 10, 15, 30, 50 | 3 |
| KNN | sklearn.neighbors.KneighborsRegressor() | n_neighbors | 5, 10, 20, 40 | 5 |
|  |  | weights | 'uniform', 'distance' | 'uniform' |
|  |  | leaf_size | 5, 10, 30, 50 | 30 |
| ANN | sklearn.neural_network.MLPRegressor() | hidden_layer_sizes | 10, 50, 100, 500 | 100 |
|  |  | activation | 'logistic', 'relu' | 'relu' |
| RF | sklearn.ensemble.RandomForestRegressor() | n_estimators | 10, 50, 100, 500, 1000 | 100 |
|  |  | min_samples_split | 2, 10, 20, 40 | 2 |
|  |  | min_samples_leaf | 1, 5, 10, 20 | 1 |
| SVR | sklearn.svm.SVR() | kernel | 'linear', 'rbf', 'sigmoid' | 'rbf' |
|  |  | gamma | 'scale', 'auto' | 'scale' |
|  |  | C | 0.05, 0.5, 1, 5, 20 | 1 |
| LightGBM | sklearn.lightgbm.LGBMRegressor() | learning_rate | 0.01, 0.05, 0.1, 0.2, 0.5 | 0.1 |
|  |  | n_estimators | 10, 50, 100, 500, 1000 | 100 |
|  |  | num_leaves | 2, 5, 21, 31, 51, 100 | 31 |
|  |  | min_child_samples | 1, 5, 10, 20, 40 | 20 |

**Supplemental Table. 2.** Baseline precision when training set contains 621 samples.

|  | **LightGBM** | | | **rrBLUP** | | |
| --- | --- | --- | --- | --- | --- | --- |
|  | **DTT** | **PH** | **EW** | **DTT** | **PH** | **EW** |
| Fixed 621 testing samples | 0.635 | 0.730 | 0.457 | 0.630 | 0.728 | 0.507 |
| Fixed 621 training samples | 0.654 | 0.721 | 0.467 | 0.632 | 0.695 | 0.489 |
| Fixed sample ratio of 9:1 | 0.622 | 0.700 | 0.478 | 0.602 | 0.699 | 0.515 |
| Fixed sample ratio of 1:9 | 0.638 | 0.714 | 0.469 | 0.627 | 0.704 | 0.500 |
| Baseline precision^1^ at 10% | 0.637 | 0.716 | 0.467 | 0.623 |  | 0.503 |

^1.^ Baseline precision is the averaged value of the above four scenarios when the training set contains 621 samples, which accounts for 10% of the population.

**Supplemental Table 3.** Benchmark testing of CropGBM and rrBLUP

| **Computing time for model training** | | | | |
| --- | --- | --- | --- | --- |
|  | Number of samples (10,000 SNPs) | | | |
| Models | 1,000 | 10,000 | 50,000 | 100,000 |
| CropGBM-CPU (min) | 1 | 2 | 8 | 15 |
| CropGBM-GPU (min) | 1 | 1 | 2 | 4 |
| rrBLUP (min) | 1 | 7 | 1,028 | >>1,028 |
| **Computing time for model training** | | | | |
|  | Number of SNPs (10,000 samples) | | | |
| Models | 1,000 | 10,000 | 50,000 | 100,000 |
| CropGBM-CPU (min) | 1 | 2 | 10 | 23 |
| CropGBM-GPU (min) | 1 | 1 | 6 | 15 |
| rrBLUP (min) | 3 | 7 | 11 | 21 |
| **Memory usage (GB) for model training** | | | | |
|  | Number of samples (10,000 SNPs) | | | |
| Models | 1,000 | 10,000 | 50,000 | 100,000 |
| CropGBM | 0.4 | 4.0 | 20.2 | 40.3 |
| rrBLUP | 0.4 | 8.4 | 115.7 | >>115.7 |
| **Memory usage (GB) for model training** | | | | |
|  | Number of SNPs (10,000 samples) | | | |
| Models | 1,000 | 10,000 | 50,000 | 100,000 |
| CropGBM | 0.8 | 4.0 | 20.0 | 40.0 |
| rrBLUP | 5.0 | 8.4 | 19.0 | 33.0 |
| **Server configuration for benchmark testing** | | | | |
| CPU | Xeon (E5-2665 2.40 GHz, 8 cores) ×2 | | | |
| Memory | 128 GB | | | |
| GPU | NVIDIA GeForce GTX-P8 1080 ×4 | | | |

**Supplemental Table 4.** Functional modules of CropGBM compared with other GS tools.

|  | Genomic selection^1^ | | | | Additional analytical functions^5^ | | | | | | Data visualization function^11^ | | | | |
| --- | --- | --- | --- | --- | --- | --- | --- | --- | --- | --- | --- | --- | --- | --- | --- |
|  | Model^2^ | GPU^3^ | Speed vs. sample size^4^ | Data pre-processing^6^ | | Population structure^7^ | GWAS^8^ | Feature selection^9^ | Kinship^10^ | Genotype data^12^ | | Phenotype data^13^ | Population structure^14^ | Feature importance^15^ |  |
| CropGBM | LightGBM | √ | Linear | √ | | √ |  | √ |  | √ | | √ | √ | √ |  |
| DeepGS | CNN | √ | Linear |  | |  |  |  |  |  | |  |  |  |  |
| BGLR | Bayes |  | Exponential |  | |  |  | √ |  |  | |  |  |  |  |
| rrBLUP | MLM |  | Exponential |  | |  | √ |  | √ |  | |  |  |  |  |
| SOMMER | MLM |  | Exponential |  | |  | √ |  | √ |  | |  |  |  |  |
| BLUPF90 | MLM |  | Exponential |  | |  |  |  | √ |  | |  |  |  |  |
| ASReml | MLM |  | Exponential |  | |  |  |  | √ |  | |  |  |  |  |
| GAPIT | MLM |  | Exponential | √ | | √ |  |  | √ | √ | | √ | √ |  |  |
| KAML | MLM |  | Exponential |  | |  |  |  | √ |  | |  |  |  |  |
| solGS | MLM |  | Exponential |  | | √ | √ |  | √ |  | |  | √ |  |  |
| SeqBreed | MLM |  | Exponential | √ | | √ | √ |  | √ | √ | |  | √ |  |  |

**Notes: description of the 15 columns.**

1. Core function of genomic selection (GS).
2. Models or algorithms used in the GS: LightGBM: light gradient boosting machines; CNN: convolutional neural network, Bayes: Bayesian inference, MLM: mixed linear model.
3. Whether the software support GPU-enabled acceleration.
4. Whether the computing speed and memory usage is linearly or exponentially increased along with the number of samples.
5. Analytical modules or functions in addition to the core GS function.
6. Whether the software includes function of data pre-processing, such as phenotype standardization, genotype recoding etc
7. Whether the software includes function of population structure analysis. For example, LightGBM includes PCA, t-SNE and OPTICS algorithms to analyse the structure of a population.
8. Whether the software includes GWAS analysis function. LightGBM doesn’t perform GWAS analysis its self. When phenotype prediction is accomplished, the users may select GWAS tools based on their choice.
9. Whether the software includes the function of feature selection, so that the SNPs with high effects may be select for marker panel design.
10. Whether the software support computation of kinship matrix.
11. The data visualization modules that facilitate users to better understand the characteristics of their data and population.
12. Genotype data visualization module plots figures related to the statistics of genotypes data, such as the distribution of allele frequency, allele heterozygosity, sample missing rate, genotype missing rate etc.
13. Phenotype data visualization module plots figures related the distribution of phenotypes.
14. Population structure visualization modules plots PCA, t-SNE, K-means and OPTICS clustering figures.
15. Feature importance visualization modules plots heatmap of high-effective SNPs selected by Information Gain (IG) scores.

**Supplemental Table 5.** The *p* values of statistical tests used for comparison in the corresponding figures

| **Fig. 1c (MLs vs. rrBLUP)** | | | | |
| --- | --- | --- | --- | --- |
|  |  | DTT | PH | EW |
| Comparison of five ML models with rrBLUP | GB vs. rrBLUP | 0.99120 | 0.84117 | 0.14208 |
|  | KNN vs. rrBLUP | 0.51123 | 0.45464 | 0.02319 |
|  | MLP vs. rrBLUP | 0.20517 | 0.46501 | 0.04942 |
|  | RF vs. rrBLUP | 0.08609 | 0.17830 | 0.00027 |
|  | SVR vs. rrBLUP | 0.13871 | 0.27661 | 0.12763 |
| **Fig. 2b (GB vs. rrBLUP)** | | | | |
|  |  | DTT | PH | EW |
| Partitions of training and testing samples | M and P | 0.00025 | 0.05092 | 0.64380 |
|  | M only | 0.70127 | 0.15315 | 0.29875 |
|  | P only | 0.36326 | 0.39680 | 0.02185 |
|  | Neither | 0.59158 | 0.09252 | 0.83061 |
| **Fig. 2c (GB vs. rrBLUP)** | | | | |
|  |  | DTT | PH | EW |
| Between populations | Jing724 F_1_ | 0.78220 | 0.72030 | 0.31450 |
|  | Zheng58 F_1_ | 0.91160 | 0.88550 | 0.28730 |
| **Fig. 3a (GB variants vs. rrBLUP)** | | | | |
|  |  | DTT | PH | EW |
| Comparisons of GB variants with rrBLUP | LGB vs. rrBLUP | 0.31890 | 0.45280 | 0.14580 |
|  | CB vs. rrBLUP | 0.61940 | 0.69490 | 0.43940 |
|  | GB vs. rrBLUP | 0.93610 | 0.97510 | 0.96620 |
|  | XGB vs. rrBLUP | 0.94880 | 0.97510 | 0.98640 |
| **Fig. 3c (rrBLUP vs. LightGBM and rrBLUP+ vs. LightGBM+)** | | | | |
|  |  | DTT | PH | EW |
| Comparison of precision when fixed effects added | Jing724 | 0.39420 | 0.28000 | 0.68820 |
|  | Jing724(+) | 1.00000 | 0.89440 | 0.57940 |
|  | Zheng58 | 0.50500 | 0.46910 | 0.37040 |
|  | Zheng58(+) | 0.84390 | 0.93290 | 0.55090 |
| **Fig. 4a (LightGBM vs. rrBLUP, fixed testing set)** | | | | |
|  |  | DTT | PH | EW |
| Training vs. testing samples | 9:1 | 0.90120 | 0.90610 | 0.96450 |
|  | 7:1 | 2.33E-14 | 1.16E-19 | 0.00161 |
|  | 5:1 | 0.00003 | 3.00E-08 | 7.43E-09 |
|  | 3:1 | 0.53317 | 0.63092 | 7.36E-15 |
|  | 1:1 | 0.23207 | 0.55809 | 1.04E-10 |
|  | 1:3 | 5.43E-07 | 9.75E-14 | 6.56E-07 |
|  | 1:5 | 4.28E-11 | 5.47E-10 | 2.07E-07 |
|  | 1:7 | 5.88E-13 | 3.34E-13 | 1.67E-08 |
|  | 1:9 | 2.77E-12 | 2.65E-16 | 1.99E-11 |
| **Fig. 4b (LightGBM vs. rrBLUP, fixed training set)** | | | | |
|  |  | DTT | PH | EW |
| Training vs. testing samples | 9:1 | 0.31049 | 0.39699 | 0.01587 |
|  | 7:1 | 0.21000 | 0.75446 | 0.03705 |
|  | 5:1 | 0.02595 | 0.08226 | 0.04093 |
|  | 3:1 | 0.00205 | 0.32229 | 0.00002 |
|  | 1:1 | 1.21E-06 | 0.03578 | 2.61E-12 |
|  | 1:3 | 1.09E-16 | 0.00001 | 6.28E-25 |
|  | 1:5 | 7.61E-23 | 3.18E-07 | 3.31E-34 |
|  | 1:7 | 5.74E-36 | 0.31049 | 2.34E-42 |
|  | 1:9 | 0.78270 | 0.92740 | 0.54030 |
| **Fig. 4c (LightGBM vs. rrBLUP, fixed ratio 9:1 training:testing)** | | | | |
|  |  | DTT | PH | EW |
| Number of total samples | 6210 | 0.00028 | 1.43E-07 | 0.02698 |
|  | 5520 | 0.00129 | 0.00467 | 0.19646 |
|  | 4830 | 0.00589 | 0.00728 | 0.48901 |
|  | 4140 | 0.23241 | 0.00933 | 0.72488 |
|  | 3450 | 0.37915 | 0.04221 | 0.77669 |
|  | 2760 | 0.71601 | 0.19094 | 0.10189 |
|  | 2070 | 0.99052 | 0.45726 | 0.08792 |
|  | 1380 | 0.42864 | 0.74553 | 0.01378 |
|  | 690 | 0.36263 | 0.95465 | 0.12514 |
| **Fig. 4d (LightGBM vs. rrBLUP, fixed ratio 1:9 training:testing)** | | | | |
|  |  | DTT | PH | EW |
| Number of total samples | 6210 | 0.00525 | 0.00086 | 5.11E-08 |
|  | 5520 | 0.00008 | 0.00004 | 7.46E-07 |
|  | 4830 | 2.79E-08 | 1.13E-08 | 0.32105 |
|  | 4140 | 4.89E-09 | 2.97E-11 | 0.74152 |
|  | 3450 | 1.02E-13 | 1.29E-11 | 0.18594 |
|  | 2760 | 0.00002 | 4.57E-07 | 0.00341 |
|  | 2070 | 5.60E-08 | 2.97E-09 | 1.66E-08 |
|  | 1380 | 1.62E-11 | 7.47E-12 | 0.00002 |
|  | 690 | 2.57E-13 | 3.31E-13 | 3.61E-11 |
| **Fig. 6a (Selected vs. Random)** | | | | |
|  |  | DTT | PH | EW |
| Number of SNPs | All | 0.99791 | 0.99779 | 0.96426 |
|  | 4K | 0.30436 | 0.81444 | 0.31636 |
|  | 3K | 0.12700 | 0.70621 | 0.13981 |
|  | 2K | 0.07750 | 0.49663 | 0.15119 |
|  | 1K | 0.01124 | 0.18808 | 0.00645 |
|  | 384 | 0.00063 | 0.03832 | 0.00279 |
|  | 192 | 0.00020 | 0.01192 | 0.00005 |
|  | 96 | 0.00074 | 0.01002 | 0.00050 |
|  | 48 | 0.00075 | 0.00184 | 0.00185 |
|  | 24 | 0.00021 | 0.00327 | 0.00062 |
|  | 12 | 0.00104 | 0.00162 | 0.00271 |

**Fig. S1.**


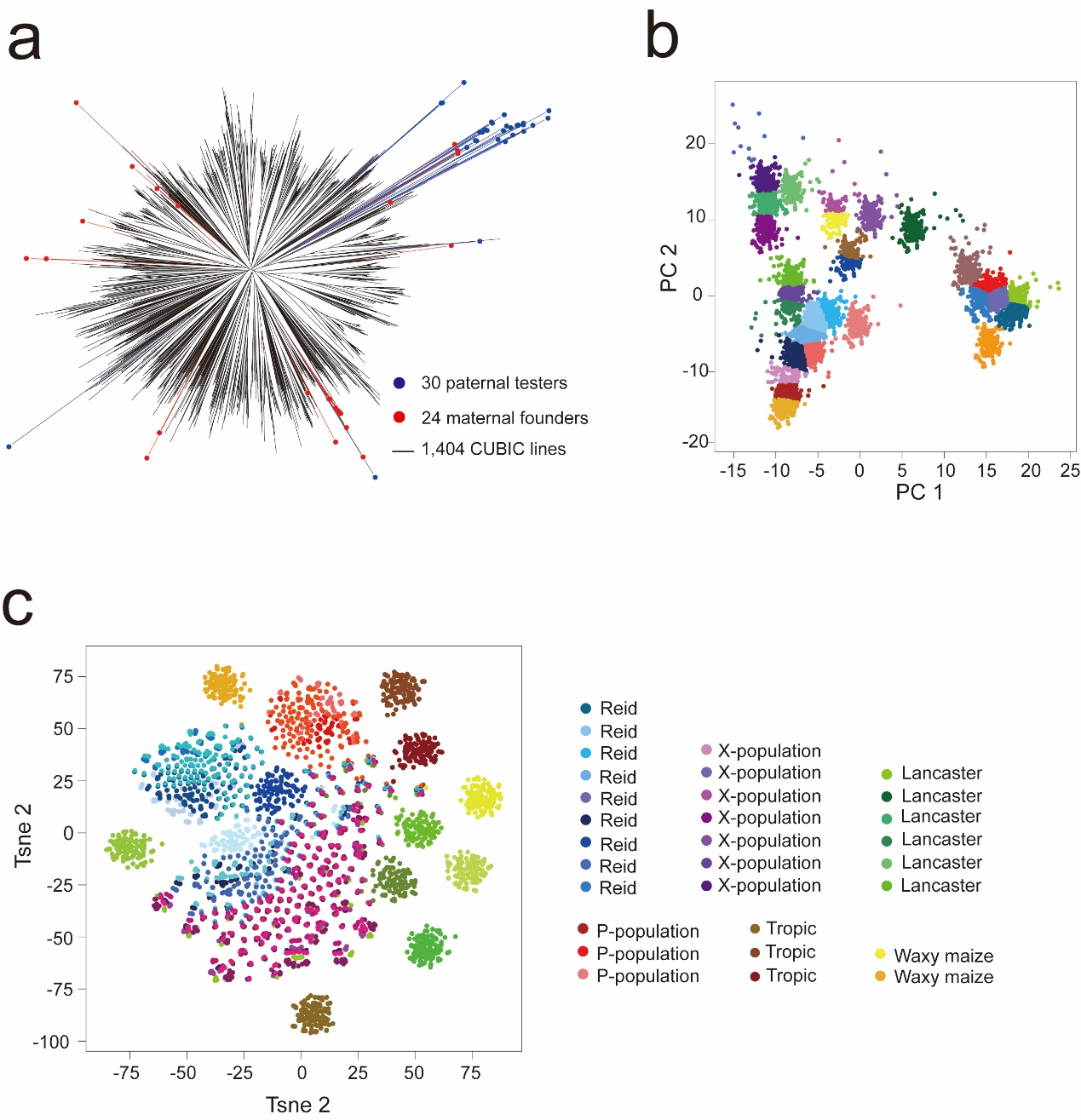


**Fig. S1.** Population structure of the 1,458 parental lines and 6,210 F_1_s visualized by phylogenetic tree, PCA, and t-SNE.

1. Phylogenetic tree of the 1,458 parental lines including 30 paternal testers, 24 maternal founders, and the 1,404 CUBIC lines.
2. Visualization of the population structure of the 6,210 F_1_s by PCA.
3. Visualization of the population structure of the 6,210 F_1_s by t-SNE algorithm. The t-SNE algorithms show more explicit separation of the thirty sets of paternal half-sibling F_1_ subpopulations from different paternal testers than the PCA algorithm.

**Fig. S2.**


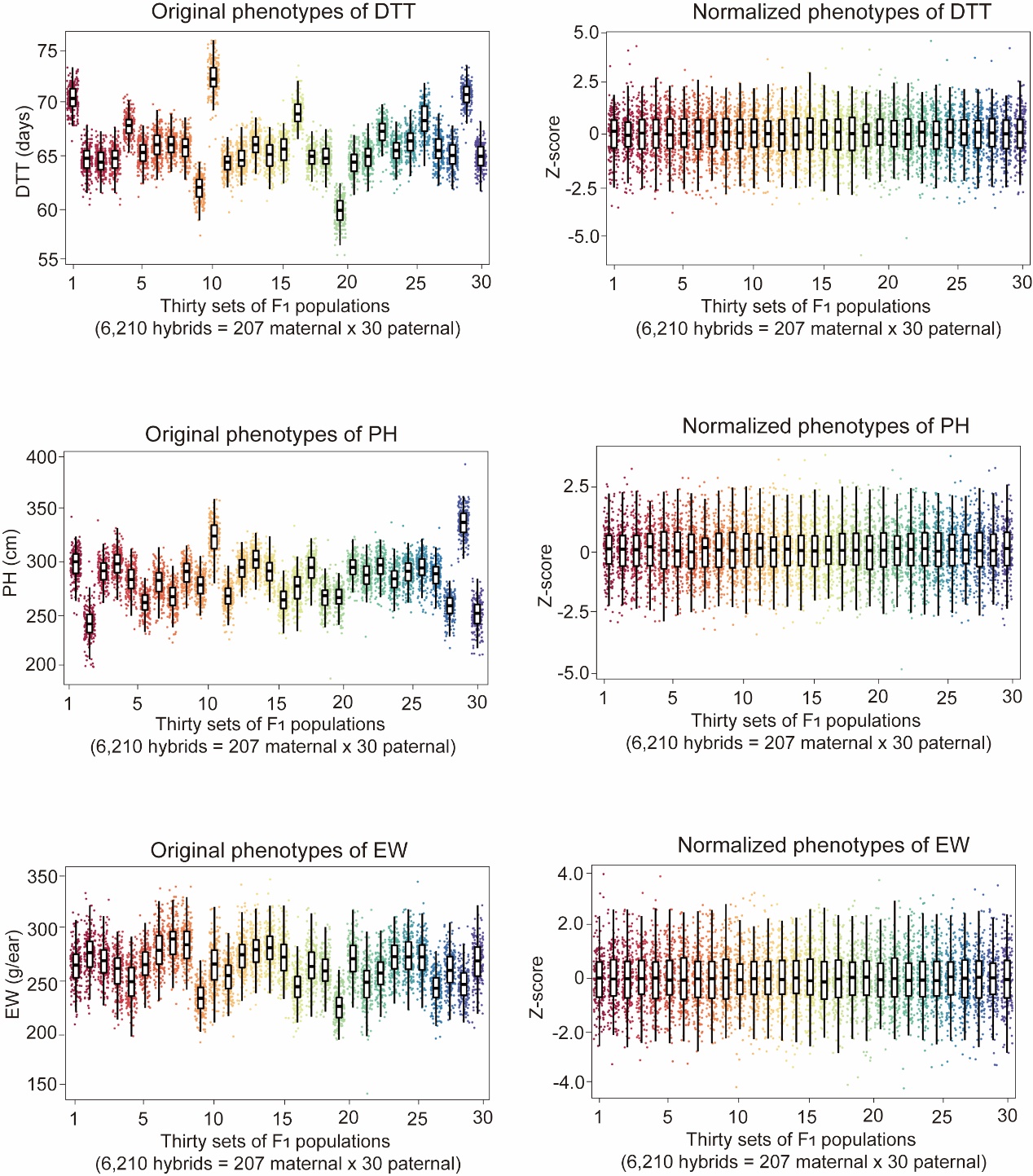


**Fig. S2**. Phenotype distribution across the thirty F_1_ populations shows between-group variations due to population stratification. Thus, z-score normalization is performed within each F_1_ population to use relative rankings to represent phenotype values.

**Fig. S3**


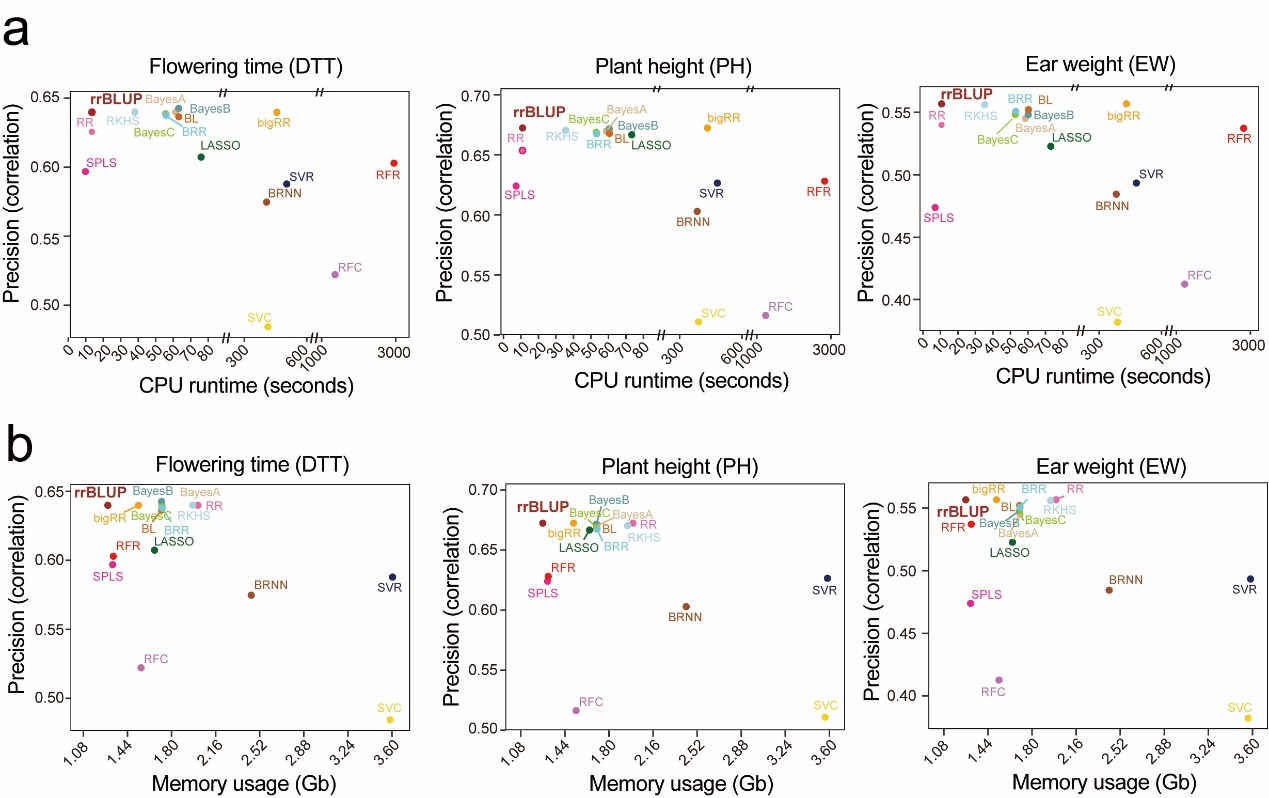


**Fig. S3**. Prediction precision and computing efficiency of rrBLUP surpassed other 15 previously published GS methods. Benchmark test dataset contains 1,428 F_1_s as samples and 32,559 SNPs as features. Configuration of the testing computer is Intel i5 CPU and 32-Gb memory.

**Fig. S4.**


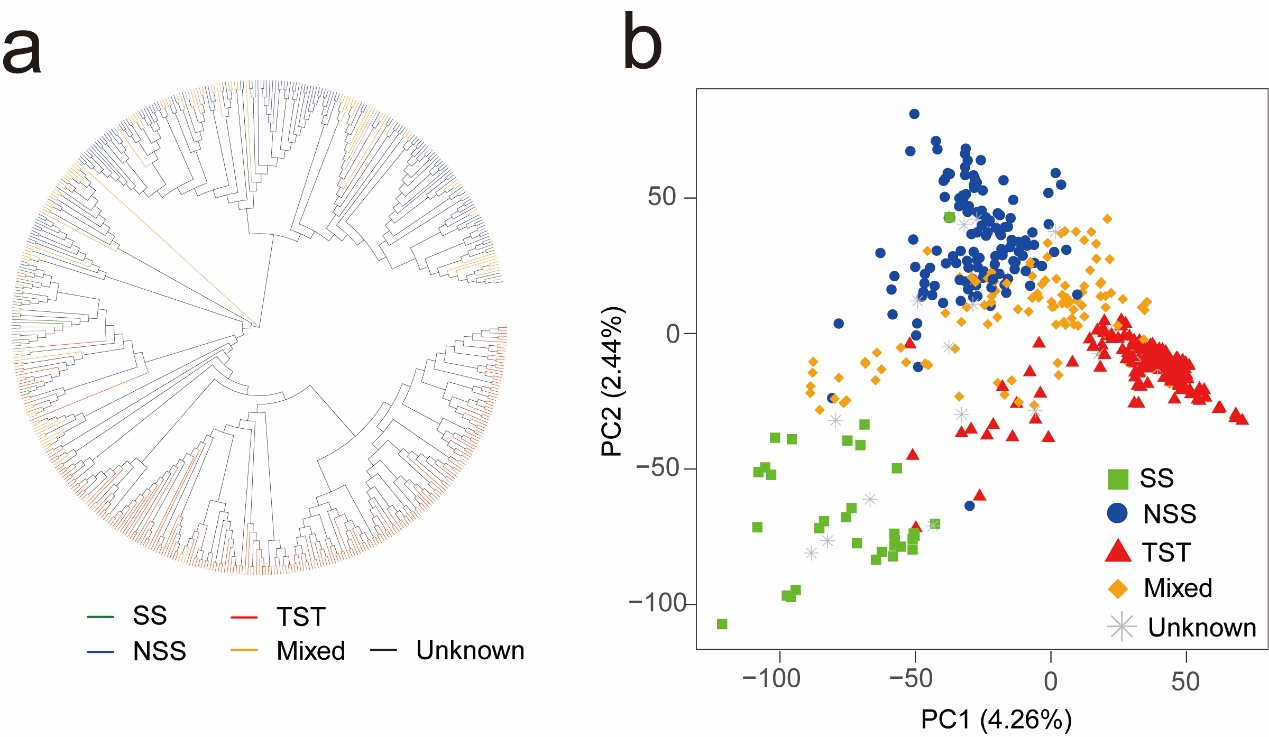


**Fig. S4. Population structure of the 527 germplasm collection.**

1. Phylogenetic tree of 527 maize lines including temperate maize of stiff straw (SS) type, non-stiff straw (NSS) type, tropic maize (TST), and maize with mixed temperate and tropic genetic background (mixed). Unclassified maize is denoted as Unknown.
2. PCA plot of the five types of maize germplasms.

**Fig. S5**


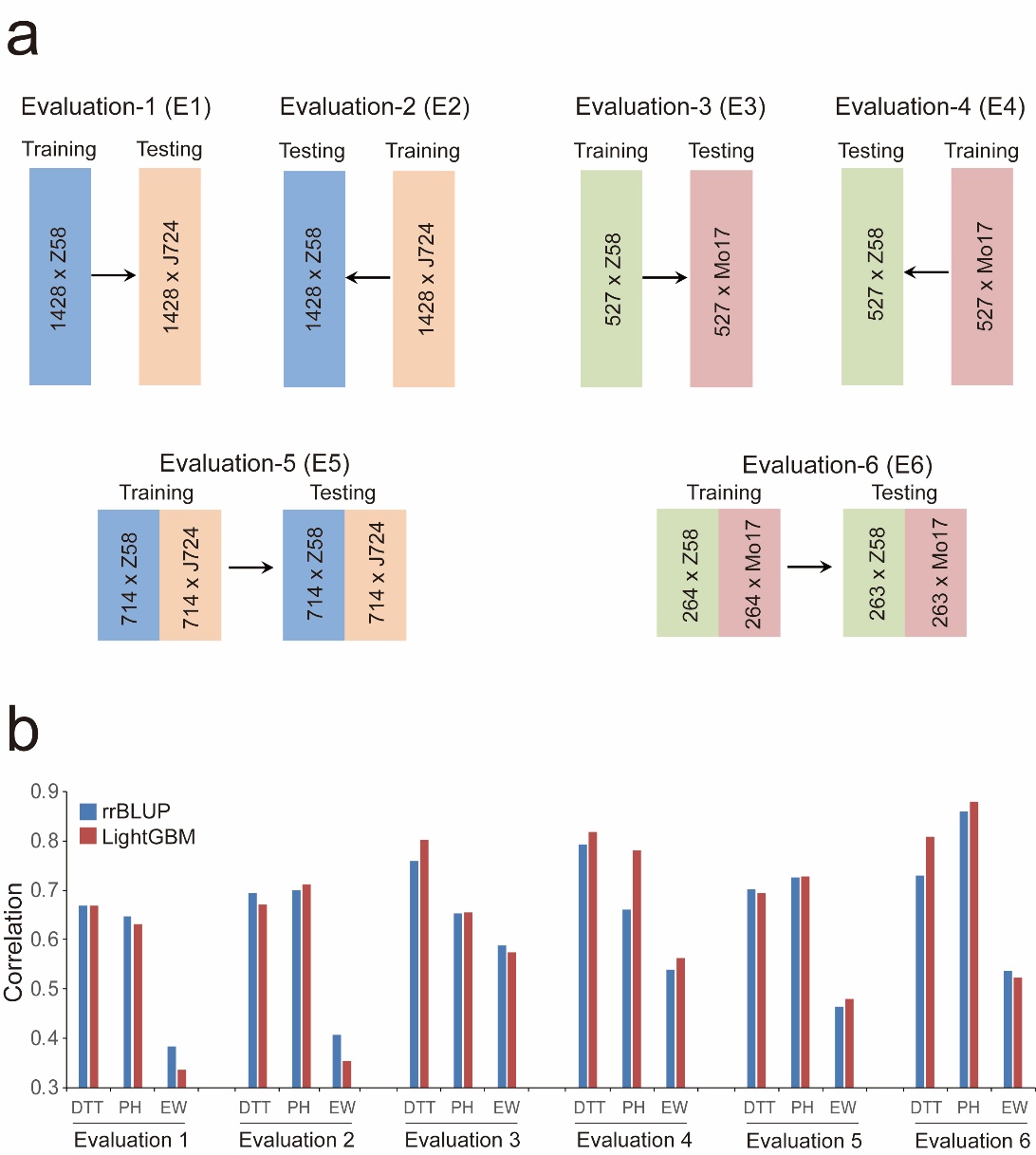


**Fig. S5. Evaluation of model stability across different populations.**

1. Six different predictive frameworks used for evaluating the model stability of rrBLUP and LightGBM. E1, E2, and E5 are the three predictive frameworks based on the crossings between 1428 CUBIC lines with Zheng58 and Jing724. E1: 1,428 Zheng58 F_1_s as the training samples to predict 1,428 Jing724 F_1_s; E2: 1,428 Jing724 F_1_s as the training samples to predict 1,428 Zheng58 F_1_s. E5: 714 Zheng58 F_1_s + 714 Jing724 F_1_s as the training samples to predict the rest 714 Zheng58 F_1_s + 714 Jing724 F_1_s. E3, E4, and E6 are the three predictive frameworks based on the crossings between 527 core germplasms with Zheng58 and Mo17. E3: 527 Zheng58 F_1_s as the training samples to predict 527 Mo17 F_1_s; E4: 527 Mo17 F_1_s as the training samples to predict 527 Zheng58 F_1_s; E6: 264 Zheng58 F_1_s + 264 Mo17 F_1_s as the training samples to predict the rest 263 Zheng58 F_1_s + 263 Mo17 F_1_s.
2. Precisions of rrBLUP and LightGBM for the three traits using the six predictive frameworks for prediction.

**Fig. S6.**


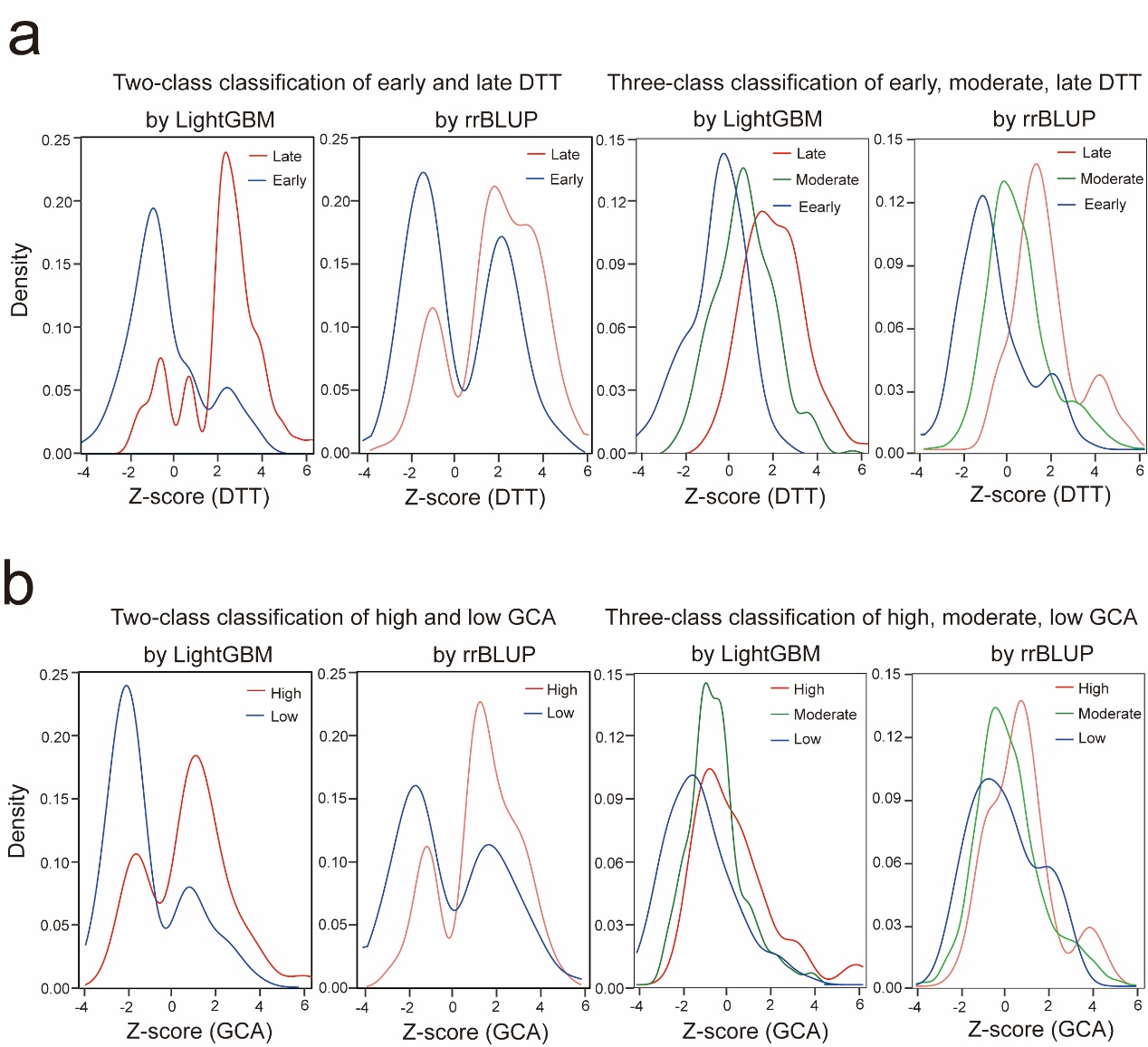


**Fig. S6. Comparison of classification precision of LightGBM and rrBLUP**

1. Distribution of Zheng58 F_1_s with early and late DTT predicted by the two-class classification function of LightGBM and rrBLUP (left two panels); and Distribution of Zheng58 F_1_s with early, moderate and late DTT predicted by the three-class classification function of LightGBM and rrBLUP (right two panels)
2. Distribution of maternal lines with high and low GCA of EW predicted by the two-class classification function of LightGBM and rrBLUP (left two panels); and Distribution of maternal lines with high, moderate, and low GCA of EW predicted by the three-class classification function of LightGBM and rrBLUP (right two panels). The computation of the general combining ability (GCA) value for each maternal line is based on the EWs of Zheng58 and Jing724 F_1_s.

**Fig. S7.**


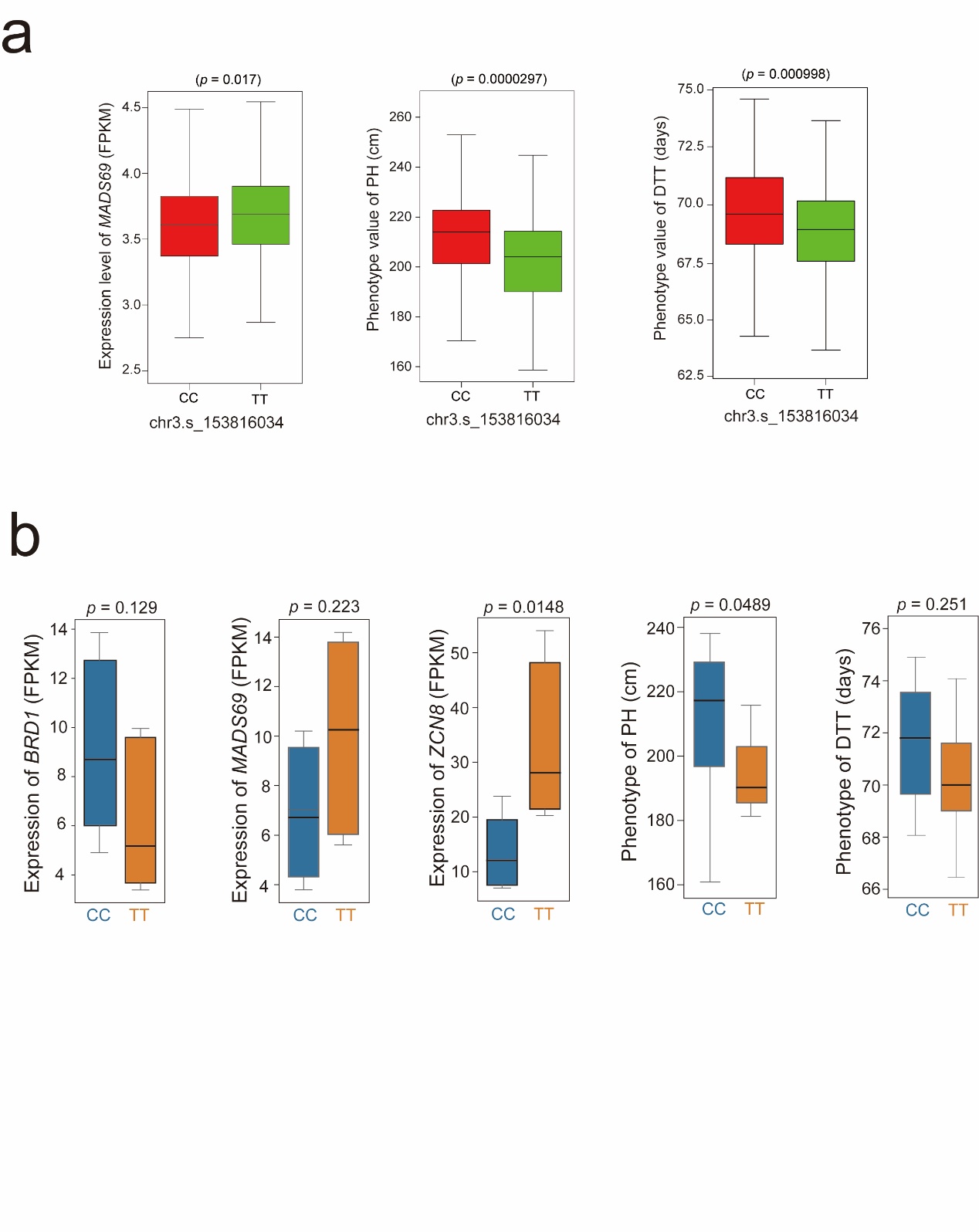


**Fig. S7. Highly-effective SNPs show correlated divergence in the genotype, phenotype, and expression of trait-associated genes.**

1. The 441 maternal lines are divided into *MADS69*-CC (124 lines) and *MADS69*-TT (317 lines) subgroups based on the genotype of chr3.s_153816034. The two subgroups exhibit significantly different expression levels of *MADS69* gene, as well as the phenotypes of DTT and PH.
2. The 23 maternal founder lines with homozygous genotypes are divided into 18 lines bearing *BRD1*-TT genotype and 5 lines bearing *BRD1*-CC genotype, based on the top SNP chr1.s_257839283 recognized by LightGBM. The *BRD1*-TT subgroup shows lower expression of *BRD1* gene, but higher expression of *ZCN8* and *MADS69*, compared with the *BRD1*-CC subgroup. Concordantly, the *BRD1*-TT subgroup shows higher PH and later DTT than the *BRD1*-CC subgroup.

**Fig. S8**


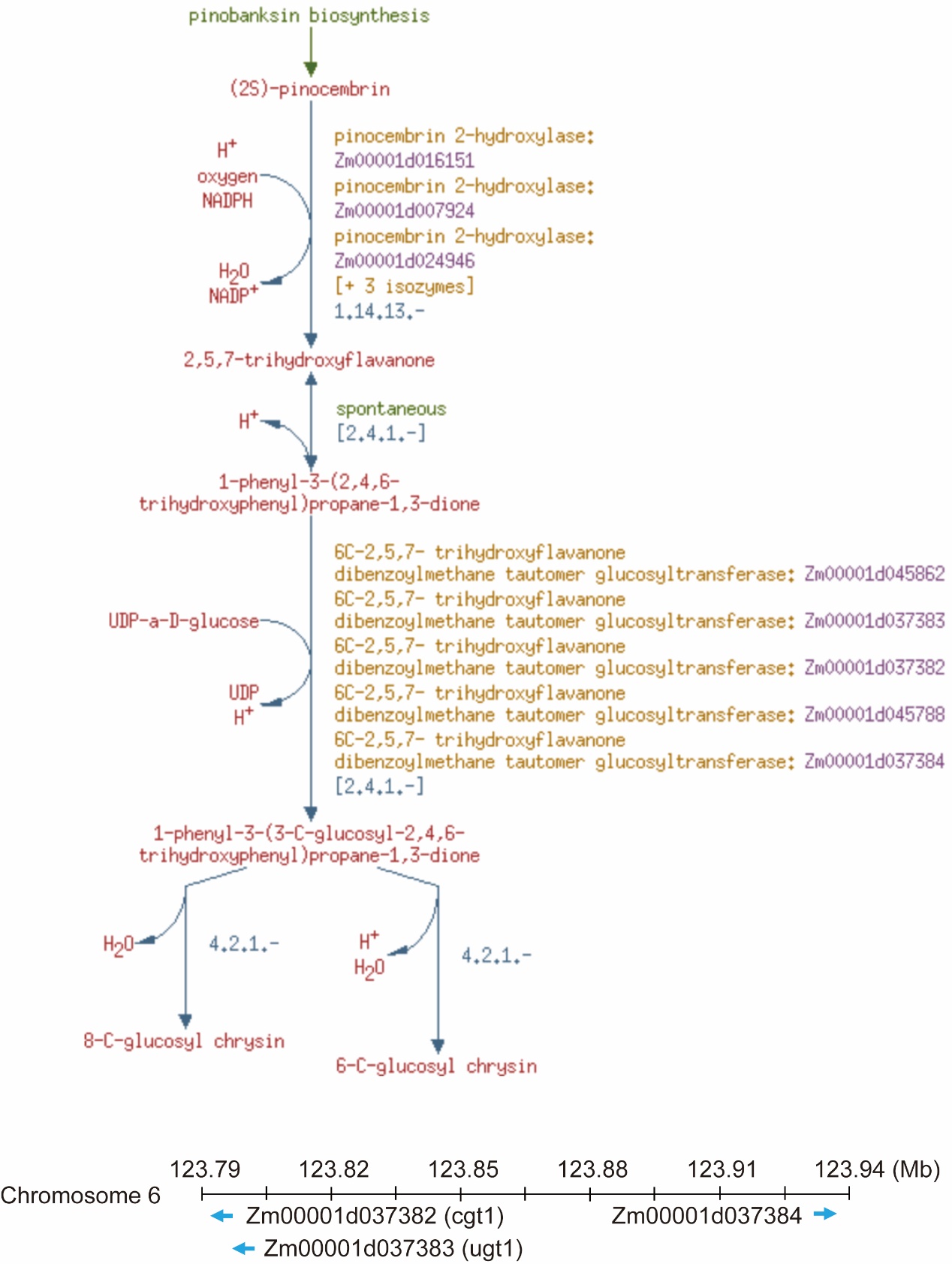


**Fig. S8.** Biosynthesis pathway of flavonoid, in which three tandemly duplicated *ZmUGT*s are identified by GWAS of the compound 3.32_594.1573n.

**Fig. S9.**


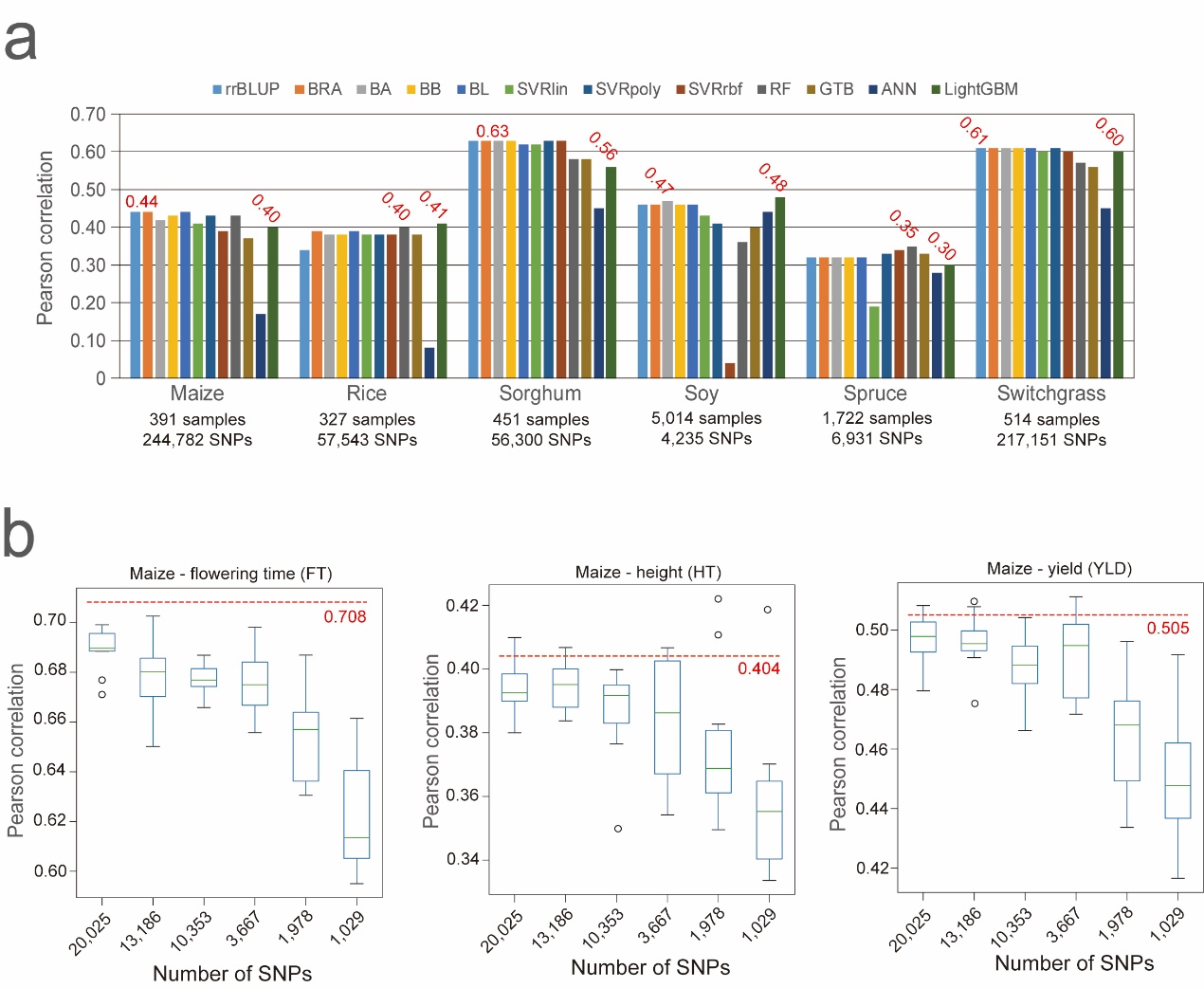


**Fig. S9**. Benchmark tests of the 14 GS models on predicting the trait of HT in six plant species.

1. Precision of LightGBM ranked as the top on method in Soy and Rice.
2. Prediction precisions of LightGBM on the traits of FT, HT and YLD using six sets of SNPs.
