# Supplementary material for: LightGBM: accelerated genomically designed crop breeding through ensemble learning
Source: Genome Biol. 2021 Sep 20;22:271. doi: 10.1186/s13059-021-02492-y (PMC8451137; doi:10.1186/s13059-021-02492-y)
Supplement: Supplementary file 2 — Additional file 2. Review history. [file 13059_2021_2492_MOESM2_ESM.docx]

Review History

**First round of review**

**Reviewer 1**

**Are you able to assess all statistics in the manuscript, including the appropriateness of statistical tests used?**

No, I do not feel adequately qualified to assess the statistics.

**Comments to author:**

This article describes an implementation of LightGBM for G2P prediction. There are several strengths. G2P prediction is a timely and important problem in agriculture. ML approaches have shown to be powerful methods for addressing this problem. The paper is well written. Major weaknesses are that I wasn't convinced that GBM (and LightGBM for that matter) is much better than rrBLUP. Also I wasn't confident of the performance that is based soly on cross validation. External test data validation is a standard these days. Finally, I was confused about the lenthy descriptions of the genes where the top SNPs reside. My understanding is that these top SNPs are linked to causal SNPs but there was no evidence indicating that they were causal SNPs. Hope these comments are useful to the authors.

**Reviewer 2**

**Are you able to assess all statistics in the manuscript, including the appropriateness of statistical tests used?**

Yes, and I have assessed the statistics in my report.

**Comments to author:**

Summary:

Yan and Xu et al. present CropGBM, a toolbox for genotype to phenotype prediction using LightGBM from Microsoft. This framework is highly efficient for large datasets, which as the authors point out, is critically important for large breeding programs. In addition to providing a command line interface for running LightGBM, CropGBM includes additional tools, like SNP filtering, trait normalization and recoding, categorial genotype recoding, population structure analysis, and exporting SNP information gain scores, all features that will be highly useful to researchers and breeders. Unfortunately, I could not effectively test the software because of an error that I would have likely needed to modify the code to overcome (see below). I also have a few concerns regarding the benchmarking of CropGBM.

Major comments:

1. The first part of the paper benchmarks models for genomic prediction. First, an initial benchmark of ML models, including GB, compared to rrBLUP (Fig. 1) and then moves to comparing rrBLUP and GB with 3 variants of GB (XGB, CatGB, LightGBM) (Fig. 2). There were a number of issues with these benchmarks:

1a. Hyperparameter optimization appears to have only been done on LightGBM (lines 509-510). Selection of optimal hyperparameters is critical for achieving peak performance, especially for ML models. So having only been included for the LightGBM, it is unsurprising that this model outperformed others.

1b. The hyperparameters used for the models (e.g. RF/GB: tree depth, # trees, # features per tree, etc. ANN: # layers, #nodes per layer, optimizer used, epochs trained, etc) have not been included in the methods and the code used to generate these predictions is not provided. One or ideally both of these would be added to ensure the benchmarking is reproducible.

1c. It is well demonstrated that no one algorithm performs best for all genomic prediction problems (Helsot 2012 Crop Sci, Blonde 2015 PLoS One, etc). While the authors demonstrate the similar and/or improved performance of GB/LightGBM using different CV methods across three traits, these comparisons are all using the same maize population. To demonstrate the range of genomic prediction problems that LightGBM is suited for, a more diverse benchmarking of its performance would be very beneficial (for example using the datasets compiled in Azodi 2019 G3). Specifically, I wonder how well LightGBM will perform on datasets where n << p and on datasets with fewer samples for training.

1d. Line 136 states that GB and rrBLUP were equivalent to the other methods for SNP based predictions, but then line 165 states that ensemble learning with GB was the optimal method based on that comparison. These two statements seem contradictory and a better justification for only moving forward with GB is needed.

1e. Statistical tests are not provided for benchmarking and other comparisons performed over many replicates (e.g. Fig 1a).

2. The conclusion that ML algorithms are more advantageous when the training samples sufficiently cover the data patterns in the testing samples (i.e. when the training/testing ratio is large) is confounded by changing the number of lines used for training. While not a major point of the paper, if the goal is to discuss the importance of training/testing ratio, then the number of training samples should be held constant and the number of testing samples should change.

3. Lines 591-593 imply that feature selection was done using IG scores calculated using the training fold data only, with performance metrics calculated from the testing data. Lines 557-560 point out that this was done 5 times to get a distribution of the precision. I just want to confirm (and suggest the authors make this point very clear when the feature selection results are first presented), that for each replicate, the IG scores are calculated on just the training data and performance on the testing data is reported. Overfitting by in some way including the testing data in feature selection process is a common issue in genomic prediction studies (Bermingham 2015 Sci Rep), I just want to make sure that is not happening here.

4. While the idea of enhancing GWAS sensitivity using predicted phenotype data is enticing, I'm concerned this approach is circular, specifically that double dipping of the data during genomic prediction and then during GWAS is leading to inflated test statistics. Could the authors comment on the limitations of such an approach or the possibility of inflated statistical strength?

5. The section of classification prediction is not particularly meaningful without a comparison. For example, how does LightGBM classification AUCROC compare to rrBLUP if the rrBLUP predicted trait values are binned into the same 2 or 3 class categories?

Code Comments:

1. I was able to follow the installation instructions provided in the CropGBM documentation. However, even after successful installation, I could not run cropgbm, because of the following error:

python ../CropGBM/cropgbm -c test_config.txt -o ../test -pg all --fileprefix genofile --fileformat ped --snpmaxmiss 0.1 --samplesmaxmiss 0.1 --maf-max 0.05 --r2-cutoff 0.7

Traceback (most recent call last):

File "../CropGBM/cropgbm", line 14, in

import Preprocessed as Pp

File "/home/ubuntu/cropGBM/CropGBM/Preprocessed.py", line 42

def exid(extract_snpid, exclude_snpid, keep_sampleid, remove_sampleid, fpf, spf, savedir， plink_path): ^

SyntaxError: invalid character in identifier

2. It would be very helpful for folks working on clusters without admin access if the cropGBM could be made available through anaconda or if a Docker/Singularity container was provided.

3. The software download link in the github README does not download CropGBM.tar.gz, I had to access the scripts by cloning the authors' Git repo.

4. Please specify that python 3 is required (lightGBM will not install with python 2).

5. The example/tutorial data doesn't have an example parameter configuration file provided and the example parameter config file shown in the documentation refers to a file that isn't in the tutorial data (e.g. 8652.phe).

6. The documentation should specify exactly what the config variables are (e.g. ppgroupfile_path), not just the class (e.g. boolean, necessary).

Minor Comments:

1. The authors wait until the discussion section to mention a benefit of GB is boosting, it would be helpful to briefly introduce this concept in the introduction. For example, around line 83.

2. Could the authors provide a brief description of the differences between the three additional GB methods (XGB, CatGB, LightGBM) and how or why those differences would impact genomic prediction?

3. Line 251: "Gradient descent of SNPs". Would recommend not using the term gradient descent here, since ANNs are a topic in this paper that term may confuse.

4. The importance of testing different CV approaches is clear in the text, but the number of different CV approaches made the results confusing and difficult to compare. CV methods #2, 3, 4, and 6 had clear reasons for being included. But why were different CV approaches used for the benchmarking (Fig 1a used method #1, Fig 2a used method #5), classification (Fig 3 used #7) and feature importance analysis (Fig 4a used #8).

5. The colors in Figure 2a and 2c are different for the same algorithms.

6. Both in Figure 2d and 2e, it is hard to see the main point (D, adding parental phenotype as a feature improves performance, and E: coding scheme doesn't make a difference), because the x-axis is sorted by method and not by trait. Sorting by trait and showing the methods with different colors would fix this.

**Reviewer 3**

**Are you able to assess all statistics in the manuscript, including the appropriateness of statistical tests used?**

Yes, and I have assessed the statistics in my report.

**Comments to author:**

The authors focused gradient boosting (GB) after comparison of several machine learning methods, and compared LightGBM, an efficient implementation of GB, with rrBLUP using real maize populations in terms of phenotype prediction. The authors also assessed its potential in GWAS or classification problems, and developed a software tool for crop breeding. The manuscript covers a broad range of analyses and the results largely seem reliable because of its large population size. Although it is unclear how much this study and the software can contribute to crop breeding because there are many studies on genomic prediction/GWAS methods and tools already published so far, the study will be suitable for publication of open journals such as Genome Biology. However, I have several concerns as listed below and hope that the authors revise following the comments.

Abstract: the current abstract does not convey the summary of the study at all. The authors should mention GB and LightGBM rather than mentioning as ensemble learning. In addition, summary of experiments, populations, and results also should be mentioned. The current version is not informative and not attractive to readers.

Figure S1 and S4: Figures representing data structure and cross-validation schemes such as Figure S1 and S4 should be included in the main text for ease of reading.

Line 113: 6210 F1 lines suddenly appear here. Brief explanations are needed to understand this without reference of Methods.

Figure S5: I cannot see the relation of this figure to Fig 1c (or difference from Fig 1c). Please add explanations.

Lines 201-206: These statements are incorrect. Adjustment with allele frequencies is required for making predicted values equivalent with breeding values (i.e., for interpretation of predicted values). If the purpose is just a prediction, adjustment with allele frequencies is not necessary even for rrBLUP (it may be obvious considering that rrBLUP is analogous to ridge regression). For example, -1, 0, 1 coding is OK for prediction with rrBLUP.

Lines 226-227 "if the trait is determined by few QTLs": Which result is this statement grounded for?

Figure 4: This is just a comment. I feel these results are interesting. GB or RF can consider the interactions between genes theoretically, but there seem to be less empirical evidence that shows this. So this is a good example and may be worth for further investigation.

Lines 317-336: I think this comparison is meaningless. Probably, the authors want to show the efficacy of data augmentation by genomic prediction in GWAS. To do so, GWAS signals should be compared between a training population and training + predicted population. In this case, comparison between 7431 samples and 7431+1221 F1 samples makes sense. The second analysis (lines 337-362) follows this principle.

Line 343: What are the 226 and 506 samples? Are these a part of Figure S1?

Lines 394-400: This would be true for the implementation that the authors used (i.e., R package rrBLUP). But situations will differ for other implementation of BLUP (or mixed models); in particular, tools developed for animal breeding (e.g., blupf90 and ASReml) are designed as memory- and time-effective because much more samples are involved in animal breeding than crop breeding. Sometimes I work on cattle breeding, but I don't use rrBLUP in such case. I don't request reanalysis, but need to mention something about other implementations.

Line 491-492: Usually, BLUEs (estimates for fixed effects), not BLUP (prediction for random effects), are used for such purpose. Please confirm.

Line 526 "29 F1 population": Probably, 29 paternal half sibs. Explicit explanations are preferred.

Figure 3: ROC usually takes the false positive rate as the x-axis. Please confirm. If the true negative rate is intendedly adopted, what is the reason?

Figure S2: small letter indices in the legend are incorrect (e to h, not a to d)

**Authors Response**

**Point-by-point responses to the reviewers’ comments:**

Reviewer #1:

This article describes an implementation of LightGBM for G2P prediction. There are several strengths. G2P prediction is a timely and important problem in agriculture. ML approaches have shown to be powerful methods for addressing this problem. The paper is well written. Major weaknesses are that I wasn't convinced that GBM (and LightGBM for that matter) is much better than rrBLUP. Also I wasn't confident of the performance that is based soly on cross validation. External test data validation is a standard these days. Finally, I was confused about the lenthy descriptions of the genes where the top SNPs reside. My understanding is that these top SNPs are linked to causal SNPs but there was no evidence indicating that they were causal SNPs. Hope these comments are useful to the authors.

1. Major weaknesses are that I wasn't convinced that GBM (and LightGBM for that matter) is much better than rrBLUP.

*Reply: We apologize that the advantages of LightGBM over rrBLUP have not been clearly stated in the first version of the manuscript. rrBLUP is undoubtedly an excellent genomic selection (GS) tool based on the benchmark test of comparing rrBLUP with the other 15 previously published GS tools (Fig. S1). Thus, rrBLUP was selected to compare with LightGBM in this work.*

*First, the motivation of developing CropGBM arose from the practical scenarios of employing GS in crop breeding. Crop germplasm is genetically diverse and usually classified to many subpopulations due to differential adaption to various ecological environments. Thus, GS precision is greatly influenced by the genetic structure of training and predicting samples, as well as the crossing scheme between different subpopulations. Prior to carrying out a GS analysis, assessment of the factors that influence GS prediction is critically important to ensure the best performance of GS. Therefore, the CropGBM toolbox is developed as a one-stop solution including SNP filtration, phenotype standardization, genotype recoding, population analysis, feature selection and then the core GS prediction. These modules are novel functions that rrBLUP doesn’t have.*

*Second, model precision and stability of LightGBM is better than that of rrBLUP when sampling rate of training data is low. Following the suggestion from Reviewer 2, we used a fixed number of testing samples (621 F1s) to compare precision of the two methods at different ratios of training vs. testing (Fig. 4). The ratio of 1:1 is a turning point from which performance of both methods dropped, but LightGBM outperformed rrBLUP in terms of precision and stability.*

*Third, LightGBM is more superior on large dataset than rrBLUP. This advantage will accommodate the growing demands in seed industry, as a company may produce hundreds of thousands of inbred lines with doubled haploid (DH) technology per year. Genotype-based selection by GS in crops is widely used nowadays. According to our benchmark testing, GPU-enabled CropGBM only spent 4 min to train the model on 100,000 samples on a desktop server (2 CPUs and 128-Gb memory). In contrast, rrBLUP spent 17 hours to train the model on 50,000 samples and failed on 100,000 samples on the same server (Tab. S3).*

*In the revised manuscript, the abovementioned advantages of CropGBM were emphasized and added in the Discussion from Line 502 to 510. We also added a supplemental table to summarize the novel features and advantages of CropGBM in comparison with the other 10 GS tools in Additional file 1: Table S4.*

2. Also I wasn't confident of the performance that is based solely on cross validation. External test data validation is a standard these days.

*Reply: We agree with the reviewer that model evaluation on external dataset is important for prediction tasks. However, a dataset for external validation may not be absolutely irrelevant to the training dataset, due to the great genetic diversity between different crop populations. In actual practice of a breeding program, GS is strictly applied on the breeding materials generated from a genetic pedigree. In another word, a GS model trained in one population will not work on another population that is genetically irrelevant to the training population.*

*However, Reviewer 2 raised a good suggestion to validate the performance of LightGBM using an external dataset compiled by Azodi et al. 2019 [1]. Detailed results from more benchmark tests with this dataset compiled from six plant species are shown in the response to Reviewer 2’s Comment 1c.*

*[1] Azodi CB, Bolger E, McCarren A, Roantree M, de Los Campos G, Shiu SH. Benchmarking Parametric and Machine Learning Models for Genomic Prediction of Complex Traits. G3 (Bethesda). 2019 Nov 5; 9(11):3691-3702.*

3. Finally, I was confused about the lengthy descriptions of the genes where the top SNPs reside. My understanding is that these top SNPs are linked to causal SNPs but there was no evidence indicating that they were causal SNPs.

*Reply: The reviewer’s understanding is correct. The top SNPs reside in the peak regions (or QTLs) of flowering time-control genes (ZCN8, RAP2.7 and MADS69) and plant height-control genes (BR2 and BRD1) are not causal variants. The top SNPs are tag SNPs linked to the trait-determinant genes in the same haplotypic blocks. Functions and causal variants of these genes have been mostly characterized and reported. RAP2.7 is a repressor of flowering time and a miniature transposon (MITE) insertion into the conserved noncoding sequence located 70 kb upstream RAP2.7 causes the downregulated expression of RAP2.7 [2]. The florigen ZCN8 is repressed by transcription factor RAP2.7, and the causal SNP is most likely a mutation occurred in the promoter of ZCN8, which influences TF binding affinity on ZCN8 [3]. The promoter and coding sequences of MADS69 are conserved, and the natural variation causing differential expression of MADS69 has not been reported yet. But the function of MADS69 in regulating flowering time is clear, based on the evidence that a Mu transposon insertion occurred in the first intron of MADS69 may cause downregulation of MADS69 expression resulting in delayed flowering time [4]. The short plant height phenotype is caused by a rare non-synonymous SNP mutation in the coding region of BR2 genes [5]. The function of BRD1 regulating plant height has been characterized using EMS induced mutants [6]. Unfortunately, the natural variation causing the differential regulation of BRD1 has not been identified.*

*Because LightGBM and rrBLUP took only 32,559 SNPs as genotype features to predict phenotype, such a low SNP density is not sufficient to identify causative mutations. But, the top SNPs with high feature importance inferred by LightGBM fall into the QTL regions of causative genes regulating the traits. The purpose of describing the genes associated with the top SNPs is to emphasize two advantages of CropGBM: first, the top SNPs inferred by feature importance analysis of LightGBM is biologically interpretable; second, this function may facilitate designing molecular markers linked to a target trait to further lower the genotyping expense. In the revised manuscript, related statement was added from Line 315 to 319.*

*[2] Liang YM, Liu Q, Wang XF, Huang C, Xu GH, Hey S, Lin HY, Li C, Xu DY, Wu LS, et al: ZmMADS69 functions as a flowering activator through the ZmRap2.7-ZCN8 regulatory module and contributes to maize flowering time adaptation. New Phytologist 2019, 221:2335-2347.*

*[3] Castelletti S, Tuberosa R, Pindo M, Salvi S. 2014. A MITE transposon insertion is associated with differential methylation at the maize flowering time QTL Vgt1. G3: Genes, Genomes, Genetics 4: 805– 812.*

*[4] Guo L, Wang X, Zhao M, Huang C, Li C, Li D, Yang C, York AM, Xue W, Xu G et al. 2018. Stepwise cis-regulatory changes in ZCN8 contribute to maize flowering time adaptation. Current Biology 28: 3005– 3015.*

*[5] Xing A, Gao Y, Ye L, Zhang W, Cai L, Ching A, Llaca V, Johnson B, Liu L, Yang X, Kang D, Yan J, Li J. A rare SNP mutation in Brachytic2 moderately reduces plant height and increases yield potential in maize. J Exp Bot. 2015 Jul;66(13):3791-802.*

*[6] Makarevitch I, Thompson A, Muehlbauer GJ, Springer NM. Brd1 gene in maize encodes a brassinosteroid C-6 oxidase. PLoS One. 2012;7(1):e30798.*

4. Is the Software likely to be of broad utility? Is it easy to install and use?

*Reply: The CropGBM is designed as a one-stop solution encompassing multiple analytical modules in addition to the core GS function. These modules can be either run individually or run as a pipeline. CropGBM is thus a toolbox covering many aspects of analyses in population genetics, association studies and ML-based data mining. Researchers in animal breeding and population genetics may also use part of the analytical modules in CropGBM. In addition, three options are provided to easier the installation of CropGBM.*

5. Does the software represent a significant advance over previously published tools (as demonstrated by direct comparison with available related software)?

*Reply: Yes. We are confident CropGBM represents a significant advance in terms of prediction precision, computing efficiency, model robustness and feature selection indicated by a series of benchmark tests. As a toolbox, CropGBM covers multiple aspects of data analyses and novel functions to facilitate the proper use of GS in crop breeding. These advantages and novel features are listed in the below response to Comment 6.*

6. Please indicate briefly the novel features and/or advantages of the software and/or please reference the relevant publications and which alternatives, if any, it should be compared with.

*Reply: CropGBM integrates multiple novel features and algorithms in addition to the core function of genomic prediction, such as supporting GPU-accelerated training, feature selection via model learning to assist marker design, dimensionality reduction and population visualization via PCA and t-SNE algorithms, sample classification via K-means and OPTICS clustering, genotype and phenotype data preprocessing and etc. These novel features are listed and compared with other tools in a new supplemental Table (Table S4) in the revised manuscript.*

7. Are the methods appropriate to the aims of the study, are they well described, and are necessary controls included?

*Reply: The methods are appropriate to the aims of the study and described clearly in the Methods. As suggested by Reviewer 2, we also added external dataset (Azodi et al. 2019) to replicate the necessary benchmark analysis. The new results are show in the revised manuscript from Line 511 to 541.*

8. Are the conclusions adequately supported by the data shown?

*Reply: Yes. We followed suggestions from Reviewer 2 to include more benchmark tests to further confirm the conclusions. Results are shown in the new Fig. 4 in the revised manuscript.*

9. Are sufficient details provided to allow replication and comparison with related analyses that may have been performed?

*Reply: Yes. The Python and R codes, and supporting data for the manuscript are all deposited in the public domain of Github, as well as the webpage of CropGBM at https://github.com/YuetongXU/Cropgbm-Paper. These resources are free to access for readers to replicate the analyses in this article.*

10. Is the paper of broad interest to others in the field, or of outstanding interest to a broad audience of biologists?

*Reply: Yes. Machine learning has been widely applied for data mining and pattern discover based on large-scale omics data in biology. Using GS prediction in maize as a case study, our article presents a standard procedure of constructing an ML system covering model selection, feature selection, model evaluation, model optimization, feature importance analysis, model interpretation, and finally, software benchmark testing. We are confident that our work may gain great popularity from the biology community to promote the use of ML to solve biological questions.*

Reviewer #2:

Major comments:

1. The first part of the paper benchmarks models for genomic prediction. First, an initial benchmark of ML models, including GB, compared to rrBLUP (Fig. 1) and then moves to comparing rrBLUP and GB with 3 variants of GB (XGB, CatGB, LightGBM) (Fig. 2). There were a number of issues with these benchmarks:

1a. Hyperparameter optimization appears to have only been done on LightGBM (lines 509-510). Selection of optimal hyperparameter is critical for achieving peak performance, especially for ML models. So having only been included for the LightGBM, it is unsurprising that this model outperformed others.

*Reply: We agree with reviewer, and performed the grid search of the optimal hyperparameter of GB, KNN, ANN, RF and SVR using the 32,559 SNPs as features. GB outperformed the other four ML models, and showed equivalent performance to rrBLUP in terms of model precision and stability. This outcome is consistent with the previous one, which is shown in the new Fig. 1c. Because using Kinship and PCA as features is not a common way in genomic selection, benchmark tests on these two types of features were removed in the revised manuscript.*

1b. The hyperparameter used for the models (e.g. RF/GB: tree depth, # trees, # features per tree, etc. ANN: # layers, #nodes per layer, optimizer used, epochs trained, etc) have not been included in the methods and the code used to generate these predictions is not provided. One or ideally both of these would be added to ensure the benchmarking is reproducible.

*Reply: The optimal hyperparameter used in Fig. 1c is included in Additional file 1: Table S1.*

1c. It is well demonstrated that no one algorithm performs best for all genomic prediction problems (Helsot 2012 Crop Sci, Blonde 2015 PLoS One, etc). While the authors demonstrate the similar and/or improved performance of GB/LightGBM using different CV methods across three traits, these comparisons are all using the same maize population. To demonstrate the range of genomic prediction problems that LightGBM is suited for, a more diverse benchmarking of its performance would be very beneficial (for example using the datasets compiled in Azodi 2019 G3). Specifically, I wonder how well LightGBM will perform on datasets where n << p and on datasets with fewer samples for training.

*Reply: We agree with the reviewer that no one algorithm performs best for all GS tasks. GS prediction is influenced by many features or factors of a breeding population or a germplasm panel, such as types of species and traits, population structure, genetic diversity, numbers of SNPs and samples, G×E effects, as well as the types of crossing schemes if the samples are hybrids involving heterosis effect. Therefore, model selection is a critical step to determine the optimal GS method for a breeding project.*

*Following the reviewer’s suggestion, we obtained the compiled dataset from Azodi et al. 2019 [1], and tested the performance of LightGBM. The test was first performed on the trait of Height (HT) across the six species. Our result showed that LightGBM ranked as the number one method in Rice and Soy (Fig. S9a). In maize, precision of LightGBM (r=0.40) on HT is lower than that of rrBLUP (r=0.44), probably attributed to the different genetic diversity of the two populations. The CUBIC population of the 1,428 inbred lines was developed from 24 elite maize lines adapted to the East and Northeast China. Field measurements of their F1 phenotypes were conducted in the same latitude range of their best-adapted “accumulated temperature belt”, in order to ensure that the phenotypes were normally expressed. Additionally, the total sample set of the F1 hybrids is composed of thirty sets of “paternal half-sibling subpopulations” which is called as “F1s” for short in the manuscript. Therefore, genetic diversity of our F1 population is much less than the 391 maize lines in the Diversity Panel which contains temperate maize, tropical maize, popcorn maize, sweet maize in America, and some exotic maize (Hirsch et al. 2014). Then, it would be a difficult job to collect the optimal phenotypes for all of the maize lines in the panel, due to the influence from G×E effects. I think the different genetic diversity of the two populations is the major reason why the precisions of the three traits (DTT=0.76, PH=0.81 and EW=0.64, situation of M&P in Fig. 2b) of our breeding population is higher than those (FT=0.71, HT=0.40 and YLD=0.51) of the Diversity Panel.*

*We included the results from the above new analysis using the compiled dataset in the six plant species, and gave the possible explanation from Line 511-530 in the Discussion part: “rrBLUP adopts maximum-likelihood algorithm to estimate fixed effects and SNP effects that are used to predicted phenotypes from genome-wide SNPs. In contrast, LightGBM scans the total feature set to select a small number of highly-effective SNPs to perform prediction. If the complexity of the population is too high, the limited number of SNPs selected by LightGBM may not sufficiently quantify all the genetic variations contributing to the trait, thus lowering the prediction precision.”*

*In my opinion, determination of a proper threshold to select superior line or F1 combination is an essential step in actual practice of GS-assisted maize breeding, because breeders care more about if the “outliers” samples, for example the top 5% of yield, can be picked out rather than the overall precision (correlation) of a model. According to my experience, an easy way is to include the genotypes of one or multiple check varieties (CKs), usually elite commercial cultivars, as spike-in samples to the predicting sample set. Then, the predicted phenotypes of CKs may be used as threshold to select superior lines or F1s whose predicted phenotypes surpass the CKs. This is by far the easiest way to overcome many shortcomings in GS breeding and help adjust inestimable non-genetic effects.*

*The above statement is just my personal view on “no one algorithm performs best for all genomic prediction problems”, and raise a possible solution to this issue in actual breeding practice. Because it’s beyond the scope of this research, I prefer to only including it in the Response Letter. Readers of Genome Biology puzzled by this issue may also access the Response letter.*

*To answer the second question related to “n << p (sample number is far smaller than feature number)”, we used six different sets of SNPs selected from the total 244,782 SNPs to run LightGBM on the 391 maize lines (Fig. S9b). Selection of SNPs followed the criteria: MAF (minor allele frequency) of a SNP is between 0.15 and 0.35; in every genomic interval of 10-Kb, 50-Kb, 100-Kb, 500-Kb, 1-Mb and 2-Mb, one SNP is randomly selected. The six sets contained 20,025 (10-Kb), 13,186 (50-Kb), 10,353 (100-Kb), 3,667 (500-Kb), 1978 (1-Mb) and 1029 (2-Mb) SNPs. Then, we performed LightGBM prediction using the six sets of SNPs, followed by comparing the results with the precisions of the three traits (HT: 0.404, FT: 0.708 and YLD: 0.505) using the 244,782 SNPs as features by LightGBM.*

*Random selection of the SNPs in each length of genomic interval was repeated for 10 times, and cross validation was repeated for 100 times. Then, a distribution of prediction precision was generated for each set of SNPs. As for the FT trait, the average precisions of 20,025, 13,186, 10,353 and 3667 SNPs are 0.689, 0.678, 0.677, 0.675, respectively, only slightly dropped compared to 0.708 using 200K SNPs; As for the HT trait, the four precisions are 0.394, 0.394, 0.386, 0.384, respectively, compared to 0.404 using all SNPs; As for the YLD trait, the three precisions are 0.497, 0.496, 0.487, 0.492, respectively, compared to 0.505. Therefore, using about 5K to 10K SNPs may still generate acceptable precisions. This amount of SNPs may not only save great amount of training times, but also a reasonable range of applying genotyping by target sequencing (GBTS) via multiplexing PCR-based GBTS or liquid chip capturing-based GBTS [7]. As I know, these two GBTS platforms only cost 5 to 10 US. dollars to genotype thousands of samples with 5K to 10K SNPs, which is an ideal solution suitable for applying GS in plants. In summary, considering both computing efficiency and genotyping expense, it would be good to reduce the amount of features with a series of model evaluation.*

*[7] Guo, Z., Wang, H., Tao, J., Ren, Y., Xu, C., Wu, K., Zou, C., Zhang, J. and Xu, Y. (2019) Development of multiple SNP marker panels affordable to breeders through genotyping by target sequencing (GBTS) in maize. Molecular Breeding 39, 1-12.*

1d. Line 136 states that GB and rrBLUP were equivalent to the other methods for SNP based predictions, but then line 165 states that ensemble learning with GB was the optimal method based on that comparison. These two statements seem contradictory and a better justification for only moving forward with GB is needed.

*Reply: We appreciate the reviewer for pointing out the unclear statement. Our purpose was to select an optimal ML method based on the comparison of five commonly used basal ML algorithms to rrBLUP (statistical model). The correct logic is that GB is equivalent to rrBLUP and GB is the optimal one among the five basal ML algorithms. Then, we moved forward to test the variants of GB (XGB, CatGB, LightGBM). The related statements were revised.*

1e. Statistical tests are not provided for benchmarking and other comparisons performed over many replicates (e.g. Fig 1a).

*Reply: Because of the limited space on the figures, it’s impossible to add p values for all comparison on the figures. But we provided a Table (Additional file 1: Table S5) containing the p values of the statistical tests used for comparison in the corresponding figures. They are Fig. 1c (MLs vs. rrBLUP), Fig. 2b (GB vs. rrBLUP), Fig. 2c (GB vs. rrBLUP), Fig. 3a (GB variants vs. rrBLUP), Fig. 3c (rrBLUP vs. LightGBM and rrBLUP+ vs. LightGBM+), Fig. 4a to 4d (LightGBM vs. rrBLUP at each ratio for the three traits), Fig. 6a (Selected vs. Random).*

2. The conclusion that ML algorithms are more advantageous when the training samples sufficiently cover the data patterns in the testing samples (i.e. when the training/testing ratio is large) is confounded by changing the number of lines used for training. While not a major point of the paper, if the goal is to discuss the importance of training/testing ratio, then the number of training samples should be held constant and the number of testing samples should change.

*Reply: We agree with the reviewer that the previous way of CVs was not rigorous. In the revised manuscript, we designed four scenarios by fixing the numbers of training samples and testing samples, and fixing the training-to-testing ratios of 9:1 and 1:9 with different population sizes. Then, influences raising from population size, sample ratio and sampling rate of training data may be rigorously tested under the four scenarios. The new results are presented in Fig. 4 and Line 228-267.*

*Scenario 1 (Fig. 4a): while the size of testing set (621 samples) held constant, numbers of training samples varied from 5,589 to 62 at 9 ratios of training vs. testing samples. The ratio of 1:1 was a turning point from which precision and stability of LightGBM and rrBLUP both dropped. But, LightGBM significantly outperformed rrBLUP, indicating better performance of LightGBM than rrBLUP, when population size is small and training samples are much fewer than testing samples.*

*Scenario 2 (Fig. 4b): while the size of training set (621 samples) held constant, numbers of testing samples varied from 62 to 5,589. Model precisions evaluated at the 9 ratios are not significantly different for both methods, which are about 0.61, 0.73 and 0.42 for DTT, PH and EW on average, respectively.*

*Scenario 3 (Fig. 4c): while the ratio of 9:1 (training vs. testing) held constant, population size gradually decreases from 6210 to 690. With the decrease of population size, stability of LightGBM and rrBLUP drops, but precisions of the two methods are relatively equivalent.*

*Scenario 4 (Fig. 4d): while the ratio of 1:9 (training vs. testing) held constant, population size gradually decreases from 6210 to 690. With the decrease of population size, stability of both methods drops, but precision of LightGBM is significantly higher than that of rrBLUP.*

*The above analyses collectively indicate the importance of sampling rate of training data in terms of determining the baseline of prediction precision, which is a critical factor shall be assessed in advance, when designing a GS project for a designated population. When the size of training set is fixed at 621 samples, which accounts for 10% of the population, the baseline precisions for DTT, PH and EW are 0.637, 0.716 and 0.467 averaged from the results of LightGBM prediction in the four scenarios. We may also conclude that LightGBM may outperform rrBLUP, when the sampling rate is lower than 10% and the size of training samples is much smaller than that of testing samples.*

3. Lines 591-593 imply that feature selection was done using IG scores calculated using the training fold data only, with performance metrics calculated from the testing data. Lines 557-560 point out that this was done 5 times to get a distribution of the precision. I just want to confirm (and suggest the authors make this point very clear when the feature selection results are first presented), that for each replicate, the IG scores are calculated on just the training data and performance on the testing data is reported. Overfitting by in some way including the testing data in feature selection process is a common issue in genomic prediction studies (Bermingham 2015 Sci Rep), I just want to make sure that is not happening here.

*Reply: We are grateful to the reviewer for pointing out a mistake. Our previous analysis did not absolutely exclude the influence from testing data, as the IGs were computed after CV. We have redone this part of analysis following the new procedure described in the Method from Line 675-684: “To exclude the influence of SNP effects from testing samples, the IGs were first computed by LightGBM in each time of sampling of 80% lines as training set, and the top 12, 24, 48, 96, 192, 384, 1000, 2000, 3000, 4000 SNPs were selected, which were based on ranking the summed IGs from high to low, as features to retrain LightGBM. The model was then used to predict phenotypes of the rest 20% lines. The sampling was repeated for 30 times to generate 30 Pearson correlations used to plot precision distribution for each set of SNPs. Meanwhile, the same procedure was applied on the same number of SNPs randomly sampled from the 32,559 SNPs to plot precision distribution as the contrast. At last, rrBLUP was performed on the 1428 maternal lines using 32,559 SNPs to compute baseline precisions for the three traits.” The new result is presented in Fig. 6a.*

4. While the idea of enhancing GWAS sensitivity using predicted phenotype data is enticing, I'm concerned this approach is circular, specifically that double dipping of the data during genomic prediction and then during GWAS is leading to inflated test statistics. Could the authors comment on the limitations of such an approach or the possibility of inflated statistical strength?

*Reply: We implemented this idea based on the fact that the algorithm of LightGBM used on genomic prediction is different from the mixed linear model (MLM) used in the GWAS tool GEMMA. If the prediction was performed by rrBLUP and GWAS by GEMMA which are both based on MLM, the “circular” issue may possibly exist. However, we cannot rule out the possibility of overestimating the QTL effects. Therefore, this method is only applicable to a rough mapping of an interested QTL in an un-phenotyped population. When the QTL location is determined, we may further compare the haplotypes of the QTLs in training and predicting populations. Limitation was described to the revised manuscript from Line 411 to 416.*

5. The section of classification prediction is not particularly meaningful without a comparison. For example, how does LightGBM classification AUCROC compare to rrBLUP if the rrBLUP predicted trait values are binned into the same 2 or 3 class categories?

*Reply: We followed the reviewer’s suggestion, and ran rrBLUP on the same dataset used to test the classification function of LightGBM. The results showed that LightGBM outperformed rrBLUP (Fig. S6).*

Code Comments:

1. I was able to follow the installation instructions provided in the CropGBM documentation. However, even after successful installation, I could not run cropgbm, because of the following error: ……

*Reply: This bug has been fixed in the upgraded CropGBM.*

2. It would be very helpful for folks working on clusters without admin access if the CropGBM could be made available through anaconda or if a Docker/Singularity container was provided.

*Reply: Installation of CropGBM is now available via anaconda, pip and source code.*

3. The software download link in the github README does not download CropGBM.tar.gz, I had to access the scripts by cloning the authors' Git repo.

*Reply: The download link has been fixed.*

4. Please specify that python 3 is required (LightGBM will not install with python 2).

*Reply: The requirement of Python 3 has been added in README.*

5. The example/tutorial data doesn't have an example parameter configuration file provided and the example parameter config file shown in the documentation refers to a file that isn't in the tutorial data (e.g. 8652.phe).

*Reply: The parameters of the configuration file are not shown in example/tutorial data because the parameters in the configuration file are exactly the same as those in the command line. Fill in the parameter value of the example in the command line into the corresponding parameter in the configuration file, which is the parameter of the example in the configuration file. In order to facilitate the user's understanding and use, we have modified "-c, --config-path" as a non-essential parameter, and deleted this parameter in the tutorial example. The“8652.phe” problem also has been fixed.*

6. The documentation should specify exactly what the config variables are (e.g. ppgroupfile_path), not just the class (e.g. boolean, necessary).

*Reply: The documentation file has been updated.*

Minor Comments:

1. The authors wait until the discussion section to mention a benefit of GB is boosting, it would be helpful to briefly introduce this concept in the introduction. For example, around line 83.

*Reply: Discussion of the benefit of gradient boosting in comparison with RF, ANN and CNN based on previous studies has been moved to the Introduction part from Line 76 to 99.*

2. Could the authors provide a brief description of the differences between the three additional GB methods (XGB, CatGB, LightGBM) and how or why those differences would impact genomic prediction?

*Reply: The main reason of why LightGBM outperforms XGBoost and CatBoost is likely due to the different ways of growing the tree structure. LightGBM adopts the strategy of leaf-wise tree growth, while the other GB variants adopts the way of level-wise tree growth. The reason why leaf-wise is better suited for genotypic data is explained in the Discussion part from Line 470-491.*

3. Line 251: "Gradient descent of SNPs". Would recommend not using the term gradient descent here, since ANNs are a topic in this paper that term may confuse.

*Reply: “gradient descent” has been changed to “gradual decrease”.*

4. The importance of testing different CV approaches is clear in the text, but the number of different CV approaches made the results confusing and difficult to compare. CV methods #2, 3, 4, and 6 had clear reasons for being included. But why were different CV approaches used for the benchmarking (Fig 1a used method #1, Fig 2a used method #5), classification (Fig 3 used #7) and feature importance analysis (Fig 4a used #8).

*Reply: The F1 hybrid population was composed by thirty sets of paternal half-sibling subpopulations (called F1s for short) generated by a breeding program. The details of the genetic design of the dataset were described in our previous work [8, 9]. The other four CV approaches were used for different purposes.*

*In new Fig. 1c, the purpose was to select an optimal ML method exhibiting equivalent precision and stability to rrBLUP, using the 6,210 F1s generated by crossing the 207 CUBIC lines with 30 paternal testers. Because the 30 testers were selected from six major heterotic groups with great genetic diversity, the 6,210 F1s exhibited great population stratification (Fig. 1b and Figure S2). The CV (Method #1) was designed to use 29 sets of F1 as training data to predict the rest set, and this procedure was repeated for 30 times. By this type of CV, model stability across different F1 subpopulations may be evaluated.*

*In Fig. 3a, the purpose was to mainly test computing efficiency on different GB variants. We used all of the 8,652 F1s to test computing time and memory usage, when evaluating the fitting ability of the five models (Method #5). CatGB was the slowest one. If grid search of optimal hyperparameter applied, CatGB spent extremely long time. Therefore, CatGB is not suitable for industrial use, as a seed company may produce tens to hundreds of thousands of inbred lines per year by DH breeding.*

*In Fig. 5, the purpose was to test the ability of classification by LightGBM, and this test was performed on the 1,428 inbred lines. Designing of this CV (Method #7) was based on the actual breeding practice. Usually, breeders categorize their breeding materials as early-flowering or late-flowering lines, then the optimal latitudes (or the “accumulated temperature belts” called by breeders) will be selected for the lines with best-adapted photoperiods and temperature. Selection of high-yield lines follows the similar way. Breeders decide which maternal lines may be promoted to advanced trials by selecting the lines featuring high GCA (general combining ability) based on its crossings with multiple paternal testers.*

*In Fig. 6a, the purpose of feature importance analysis is to facilitate the design of a panel of trait-associated makers suited for low-expense genotyping solution, such as the GBTS (genotyping by targeted sequencing) platform. Feature importance analysis was carried out in the 1,428 maternal lines homozygous genotypes (Method #8). To evaluate the effectiveness of feature selection by LightGBM (Fig. 6a), sampling of 80% of the 1428 maternal lines were repeated for 30 times, and IGs were computed at each time of repeat to select highly-effective SNPs to retrain LightGBM, followed by predicting phenotypes of the rest 20% lines.*

*Designing of the above four CV approaches was mostly based on the needs in actual breeding practice, and thus it’s difficult to cite literature to support these reasons. So, if the reviewer is not strongly against our choice, we prefer to only keeping these explanations in this Response Letter which is also published as online Additional File in Genome Biology.*

*[8] Liu HJ, Wang XQ, Xiao YJ, Luo JY, Qiao F, Yang WY, Zhang RY, Meng YJ, Sun JM, Yan SJ, et al: CUBIC: an atlas of genetic architecture promises directed maize improvement. Genome Biology 2020, 21.*

*[9] Xiao, Y., Jiang, S., Cheng, Q., Wang, X., Yan, J., Zhang, R., Qiao, F., Ma, C., Luo, J. and Li, W. (2021) The genetic mechanism of heterosis utilization in maize improvement. Genome Biology 22, 1-29*

5. The colors in Figure 2a and 2c are different for the same algorithms.

*Reply: We changed the color of rrBLUP to orange.*

6. Both in Figure 2d and 2e, it is hard to see the main point (D, adding parental phenotype as a feature improves performance, and E: coding scheme doesn't make a difference), because the x-axis is sorted by method and not by trait. Sorting by trait and showing the methods with different colors would fix this.

*Reply: We followed the reviewer’s suggestion and revised the Figure.*

Reviewer #3:

The authors focused gradient boosting (GB) after comparison of several machine learning methods, and compared LightGBM, an efficient implementation of GB, with rrBLUP using real maize populations in terms of phenotype prediction. The authors also assessed its potential in GWAS or classification problems, and developed a software tool for crop breeding. The manuscript covers a broad range of analyses and the results largely seem reliable because of its large population size. Although it is unclear how much this study and the software can contribute to crop breeding because there are many studies on genomic prediction/GWAS methods and tools already published so far, the study will be suitable for publication of open journals such as Genome Biology. However, I have several concerns as listed below and hope that the authors revise following the comments.

1. Abstract: the current abstract does not convey the summary of the study at all. The authors should mention GB and LightGBM rather than mentioning as ensemble learning. In addition, summary of experiments, populations, and results also should be mentioned. The current version is not informative and not attractive to readers.

*Reply: The Abstract has been rewritten in accordance with the requirement (word limitation: 100) of a Method article on Genome Biology.*

2. Figure S1 and S4: Figures representing data structure and cross-validation schemes such as Figure S1 and S4 should be included in the main text for ease of reading.

*Reply: Figure S1 (data structure of 8,652 F1) was integrated to Fig. 1 (Fig. 1a), and Figure S4 (four schemes of CV) was integrated to Fig. 2 (Fig. 2a).*

3. Line 113: 6210 F1 lines suddenly appear here. Brief explanations are needed to understand this without reference of Methods.

*Reply: We described the genetic design of the 6,210 F1 hybrids and explained the genetic complexity of the 30 sets of paternal half-sibling subpopulations in the first section of Results (Line 111 to 115; 123 to 129).*

4. Figure S5: I cannot see the relation of this figure to Fig 1c (or difference from Fig 1c). Please add explanations.

*Reply: Fig. 1c included the situation of neither maternal or paternal genotypes (Neither) appeared in the training set. Figure S5 included the situation of both maternal and paternal genotypes (M and P) appeared in the training set. In the new Fig. 2b, the situation of “M and P” was added, and the original Figure S5 was deleted.*

5. Lines 201-206: These statements are incorrect. Adjustment with allele frequencies is required for making predicted values equivalent with breeding values (i.e., for interpretation of predicted values). If the purpose is just a prediction, adjustment with allele frequencies is not necessary even for rrBLUP (it may be obvious considering that rrBLUP is analogous to ridge regression). For example, -1, 0, 1 coding is OK for prediction with rrBLUP.

*Reply: We agree with the reviewer that these statements are not the correct reason for converting the ten states of genotypic characters to ten digits (0-9) by LightGBM. A better reason is that this function is a convenient way of converting multi-allelic SNPs (e.g., A >> G, T) to digital features. Because the coding schemes of multi-allelic SNPs in rrBLUP is complicated, the usual way is to only keep bi-allelic SNPs for GS prediction. If applying GS on a polyploidy plant whose genome contains a substantial fraction of multi-allelic SNPs, the 0-9 recoding function in CropGBM will greatly easier the step of genotype data processing. We revised the related statements explaining the advantage of 0-9 genotype coding scheme in the revised manuscript (Line 212-226).*

6. Lines 226-227 "if the trait is determined by few QTLs": Which result is this statement grounded for?

*Reply: This statement was based on the general assumption that classification analysis is usually suitable to qualitative traits determined by single gene or QTLs with major effects. This statement is not applicable to the trait of DTT and EW. So, this sentence was removed.*

7. Figure 4: This is just a comment. I feel these results are interesting. GB or RF can consider the interactions between genes theoretically, but there seem to be less empirical evidence that shows this. So this is a good example and may be worth for further investigation.

*Reply: Yes, we agree with the reviewer’s assumption. The top SNPs with high IGs recognized by LightGBM are the nodes of a decision tree. The genotype of a top SNP associated to a trait may then be used to divide the total samples into two subgroups of samples (or two child nodes). In each child node, different top SNPs may be further recognized by LightGBM to indicate their associations to different sets of genes in the two subgroups of samples. Then, the genes in the parent and child nodes may presumably interact with each other. The structure of a decision tree representing the relationships of parent and child nodes generated by LightGBM, may theoretically reflect the interactions of a gene network. It should be noted that, the term of “interaction” here is defined in the perspective of quantitative genetics. Whether direct or indirect interactions occur between the genes based on the definition of “interaction” in molecular biology, namely gene expression regulation or protein-protein interaction, require experimental validations.*

*This idea is actually supported by our recently published work using the same 1,428 CUBIC lines, in which the gene BRD1 was identified as a major-effect gene determining the trait PH [9]. Based on the genotype (CC and TT) of the peak SNP associated with BRD1, the 1,428 lines may be divided into the BRD1-CC and BRD1-TT subgroups containing 584 and 834 lines, respectively (Figure 6). The two subgroups of samples exhibited different peaks associated with different genes. It potentially suggests that different set of genes may function under different genotypic background of BRD1 gene, which refers to “genetic interaction”. If the population is large enough, it’s possible for LightGBM to reconstruct a gene interaction network from the structure of a decision tree. Therefore, the idea raised by the reviewer is definitely worthy of further investigation in future.*

*[9] Xiao, Y., Jiang, S., Cheng, Q., Wang, X., Yan, J., Zhang, R., Qiao, F., Ma, C., Luo, J. and Li, W. (2021) The genetic mechanism of heterosis utilization in maize improvement. Genome Biology 22, 1-29.*

8. Lines 317-336: I think this comparison is meaningless. Probably, the authors want to show the efficacy of data augmentation by genomic prediction in GWAS. To do so, GWAS signals should be compared between a training population and training + predicted population. In this case, comparison between 7431 samples and 7431+1221 F1 samples makes sense. The second analysis (lines 337-362) follows this principle.

*Reply: Yes. We want to show that adding samples with predicted phenotypes to training set may enlarge the population size, resulting in enhanced GWAS signals. This may facilitate gene discovery in an un-phenotyped population. However, the QTL effect might be overestimated as concerned by Reviewer 2. As suggested by the Reviewer 2, we discussed the limitations of applying such approach. We also followed the reviewer’s suggestion to compare GWAS signals between training population and training + predicting population shown in New Fig. 7.*

9. Line 343: What are the 266 and 509 samples? Are these a part of Figure S1?

*Reply: The 266 and 509 samples are not part of the 8,652 F1 hybrids. The population of the 775 (266 + 509) inbred lines is a core germplasm collection of temperate maize. While the metabolomes of the 266 lines have been profiled at seedling stage with salt treatment, the 509 lines haven’t due to the high expense of metabolome profiling. The metabolomic data of the 266 lines have been recently published [10].*

*[10] Liang X, Liu S, Wang T, Li F, Cheng J, Lai J, Qin F, Li Z, Wang X, Jiang C. Metabolomics-driven gene mining and genetic improvement of tolerance to salt-induced osmotic stress in maize. New Phytologist. 2021. 230(6): 2355-2370.*

10. Lines 394-400: This would be true for the implementation that the authors used (i.e., R package rrBLUP). But situations will differ for other implementation of BLUP (or mixed models); in particular, tools developed for animal breeding (e.g., blupf90 and ASReml) are designed as memory- and time-effective because much more samples are involved in animal breeding than crop breeding. Sometimes I work on cattle breeding, but I don't use rrBLUP in such case. I don't request reanalysis, but need to mention something about other implementations.

*Reply: We tested BLUPF90 using 25,000 samples on the same server, because 25,000 is the maximum amount of genotyped samples that BLUPF90 can take. On the same dataset containing 25000 samples and 10,000 SNPs, BLUPF90 spent 45 minutes and consumed 36 GB memory; and LightGBM spent 5 minutes and consumed 10 Gb memory. The result of this test is included in the revised manuscript. Because ASReml is commercial software and we do not have the license, we were not able to test ASReml. Because of the wide application of DH breeding and vastly reduced expense of genotyping by GBTS, a seed company may produce tens of thousands inbred lines per year, as well large amount of genotype and phenotype data F1 hybrids per year. We are confident that CropGBM may gain great popularity in crop breeding in the near future.*

11. Line 491-492: Usually, BLUEs (estimates for fixed effects), not BLUP (prediction for random effects), are used for such purpose. Please confirm.

*Reply: We are sure that BLUP was used to compute the phenotypic values of each F1 individual by treating “genotypes” as random effects. As the statement of “To reduce the influence of the environment” may cause confusion, this sentence was removed.*

12. Line 526 "29 F1 population": Probably, 29 paternal half sibs. Explicit explanations are preferred.

*Reply: The reviewer is correct. The full name of each subpopulation of F1 hybrids generated by crossing 207 maternal lines with one paternal tester should be called a “paternal half-sibling population” and we used “F1s” for short. We added explanation of the genetic design of the 30 sets of F1s in the revised manuscript (Line 119 to 121).*

13. Figure 3: ROC usually takes the false positive rate as the x-axis. Please confirm. If the true negative rate is intendedly adopted, what is the reason?

*Reply: We thank the reviewer for pointing out the mistake. The X-axis is false positive rate and we revised the Figure.*

14. Figure S2: small letter indices in the legend are incorrect (e to h, not a to d).

*Reply: This mistake is corrected in the revised manuscript.*

**Second round of review**

**Reviewer 2**

The updated manuscript and response to reviewers from Yan and Xu et al. thoroughly addressed my original comments. Taking care to remove overfitting during the feature selection process and the extension of the training/testing ratio analysis improved the rigor of the study from my view. Further, by including plant height predictions in 6 additional species, the authors provide useful insight into the strengths and weaknesses of LightGBM. While these results are ultimately less unanimous than the results presented in the original manuscript, they still demonstrate that LightGBM is a powerful new tool and that it results in improved predictive performance in some cases (a more believable outcome given the complexity of genomic prediction problems). I was also now able to install LightGBM easily using conda.

Minor comment:

1. The authors state in their response to reviewers and in the methods that a grid search is now performed on all the machine learning methods to select optimal hyperparameters and thus compare the models more fairly. But Table S1 only provides the hyperparameter values used, not what values were included in the grid search, so the thoroughness of this grid search cannot be assessed.

2. While up to the authors, I would recommend three changes to how the results are presented:

a. Moving a subset of panels in Figure 4 to the supplement.

b. Moving all or part of Figure S9 to the main text.

c. Combining Figure 5 and Figure S6 by showing LightGBM results with a solid line and rrBLUP with a dashed line.

**Reviewer 3**

Thank you for nice revisions. I have no further comments.

**Authors Response**

**Point-by-point responses to the reviewers’ comments:**

Reviewer #2:

The updated manuscript and response to reviewers from Yan and Xu et al. thoroughly addressed my original comments. Taking care to remove overfitting during the feature selection process and the extension of the training/testing ratio analysis improved the rigor of the study from my view. Further, by including plant height predictions in 6 additional species, the authors provide useful insight into the strengths and weaknesses of LightGBM. While these results are ultimately less unanimous than the results presented in the original manuscript, they still demonstrate that LightGBM is a powerful new tool and that it results in improved predictive performance in some cases (a more believable outcome given the complexity of genomic prediction problems). I was also now able to install LightGBM easily using conda.

Minor comment:

1. The authors state in their response to reviewers and in the methods that a grid search is now performed on all the machine learning methods to select optimal hyperparameters and thus compare the models more fairly. But Table S1 only provides the hyperparameter values used, not what values were included in the grid search, so the thoroughness of this grid search cannot be assessed.

2. While up to the authors, I would recommend three changes to how the results are presented:

a. Moving a subset of panels in Figure 4 to the supplement.

b. Moving all or part of Figure S9 to the main text.

c. Combining Figure 5 and Figure S6 by showing LightGBM results with a solid line and rrBLUP with a dashed line.

*Reply: Following reviewer 2’s suggestion, we added a column of parameter values used for grid search, in addition to the best values selected by grid search.*

*Reviewer 2’s optional suggestion “c. Combining Figure 5 and Figure S6 by showing LightGBM results with a solid line and rrBLUP with a dashed line.” is a good idea. We revised the Figure 5 and related texts and figure legends based on his/her suggestions.*

*All the codes and dataset involved in the analysis of this paper have been deposited in GitHub: https://github.com/YuetongXU/Cropgbm-genomebiology and Zenodo: https://doi.org/10.5281/zenodo.5431934. The two links are also included in the reference list as 41 and 42.*
